# Genomic Inbreeding and Relatedness in Wild Panda Populations

**DOI:** 10.1371/journal.pone.0160496

**Published:** 2016-08-05

**Authors:** John R. Garbe, Dzianis Prakapenka, Cheng Tan, Yang Da

**Affiliations:** 1 Minnesota Supercomputer Institute, University of Minnesota, Minneapolis, Minnesota, United States of America; 2 Department of Animal Science, University of Minnesota, Saint Paul, Minnesota, United States of America; 3 State Key Laboratory for Agrobiotechnology, China Agricultural University, Beijing, China; Smithsonian Conservation Biology Institute, UNITED STATES

## Abstract

Inbreeding and relatedness in wild panda populations are important parameters for panda conservation. Habitat loss and fragmentation are expected to increase inbreeding but the actual inbreeding levels in natural panda habitats were unknown. Using 150,025 SNPs and 14,926 SNPs selected from published whole-genome sequences, we estimated genomic inbreeding coefficients and relatedness of 49 pandas including 34 wild pandas sampled from six habitats. Qinling and Liangshan pandas had the highest levels of inbreeding and relatedness measured by genomic inbreeding and coancestry coefficients, whereas the inbreeding levels in Qionglai and Minshan were 28–45% of those in Qinling and Liangshan. Genomic coancestry coefficients between pandas from different habitats showed that panda populations from the four largest habitats, Minshan, Qionglai, Qinling and Liangshan, were genetically unrelated. Pandas between these four habitats on average shared 66.0–69.1% common alleles and 45.6–48.6% common genotypes, whereas pandas within each habitat shared 71.8–77.0% common alleles and 51.7–60.4% common genotypes. Pandas in the smaller populations of Qinling and Liangshan were more similarly to each other than pandas in the larger populations of Qionglai and Minshan according to three genomic similarity measures. Panda genetic differentiation between these habitats was positively related to their geographical distances. Most pandas separated by 200 kilometers or more shared no common ancestral alleles. The results provided a genomic quantification of the actual levels of inbreeding and relatedness among pandas in their natural habitats, provided genomic confirmation of the relationship between genetic diversity and geographical distances, and provided genomic evidence to the urgency of habitat protection.

## Introduction

Understanding genetic diversity including genetic relatedness and inbreeding levels in wild panda populations is important for preserving panda genetic diversity through *ex situ* conservation and reintroduction programs. Over recent years, knowledge of the panda genetic diversity based on the analysis of molecular variants has been growing at a fast pace. A whole-genome sequence study using structure analysis and principal component analysis (PCA) classified wild pandas sampled from all six panda habitats into three genetically different groups [1] but this study did not estimate inbreeding and relatedness among individuals within and between habitats. Genetic diversity analysis of pandas from various origins using microsatellite markers generated information about the genomic representation of the wild panda population by the captive population [2,3], inbreeding and relatedness in panda captive breeding population [3], historical changes of genetic diversity in the two largest panda habitats [4], and variations of the MHC polymorphism in different habitats [5]. Current captive breeding of giant panda uses the average of coancestry (kinship) coefficients as a guiding parameter for selecting panda mates to avoid inbreeding [6]. However, the calculation of coancestry coefficients assumes unrelated wild founders and hidden inbreeding could occur if some of the founders in fact were related. A study using nineteen microsatellite markers detected the existence of substantial inbreeding and relatedness among wild-born and captive-born pandas managed at panda breeding centers [3]. Knowledge of such inbreeding and relatedness not documented in the panda pedigree should be helpful for minimizing hidden inbreeding due to related wild founders that were assumed unrelated in the calculation of inbreeding coefficients using pedigree data.

Although studies using microsatellite markers contributed to the understanding of panda genetic diversity and relationships, the small number of microsatellite markers could only cover a small fraction of the panda genome, leaving unanswered question whether the limited genome coverage could have affected the results. In contrast, single nucleotide polymorphism (SNP) markers from the panda whole-genome sequence data would offer the most complete coverage of the panda genome [1], and should provide more reliable assessment of the genetic diversity, inbreeding and relatedness in panda populations than microsatellite markers with limited genome coverage. The approach of genomic relationships using genome-wide SNP markers [7] provides an approach to study the genetic diversity of different populations at the individual level, provides an approach for estimating inbreeding and relatedness in the absence of pedigree information, and has been widely accepted for genomic selection and for assessing inbreeding and relatedness [8–14]. However, estimates of panda inbreeding and relatedness using genome-wide SNP markers have been unavailable in wild or captive populations.

Habitat size and genetic diversity are two related issues in panda conservation. Habitat loss was one of the major factors contributing to the declines of the wild panda population leading to 1988 when China’s Wildlife Protection Law was enacted [4]. The recently concluded Fourth National Panda Survey identified habitat loss and fragmentation to be the currently most serious threat to panda conservation [15]. The blockage of gene flow between fragmented populations due to habitat fragmentation and the drastically reduced population size of each fragmented population inevitably would lead to increased inbreeding, which is typically associated with reduced survival and fertility [16] and could lead to the extinction of small and isolated populations [17]. However, the actual levels of inbreeding and relatedness among wild pandas in their natural habitats have not been previously determined.

In this study, we estimated genomic inbreeding and relatedness in wild panda populations within and across their natural habitats using the approach of genomic relationship and high quality SNPs covering the entire panda genome selected from the panda whole-genome sequence data [1]. This dataset also contained location information for each panda DNA sample collected in the wild, providing an opportunity to determine the relationship between geographical distance and genomic similarity in wild pandas. Specifically, this study aimed to answer three questions: 1) inbreeding levels of wild pandas within each habitat, 2) genomic relatedness and similarity within and between habitats, and 3) the relationship between the geographical distance separating the pandas and the genomic similarity including relatedness among the wild pandas.

## Methods

### Panda sample and SNP selection from panda whole-genome sequences

The forty-nine pandas used in this study were from a published study [1], with thirty-four wild pandas, fourteen crossbreds and one panda with unknown origin. DNA samples of the thirty-four wild pandas were collected at fifteen locations in all six panda habitats, including one wild panda from Daxiangling (DXL), two from Liangshan (LS), seven from Minshan (MIN), eight from Qinling (QIN), fifteen from Qionglai (QIO), and one from Xiaoxiangling (XXL). The fourteen crossbreds included four crossbreds of MIN × LS, three crossbreds of QIO × MIN, four crossbreds of QIO × MIN, three crossbreds of QIN × QIO, and these crossbreds were from China Conservation and Research Center for the Giant Panda in Wolong, Chengdu Research Base of Giant Panda Breeding, and Beijing Zoo. The panda with unknown origin was included to utilize all available pandas for SNP filtering and selection as well as calculation of allele frequencies required for calculating genomic inbreeding coefficient, genomic relationships and similarity measures.

The resequencing reads for the forty-nine pandas deposited in the NCBI Sequence Read Archive (SRA) under accession SRA053353 were downloaded from DNA Data Bank of Japan at: ftp.ddbj.nig.ac.jp/ddbj_database/dra/fastq/SRA053/SRA053353. Trimmomatic [18] was used to trim low-quality bases and Illumina sequencing adapter sequences from the ends of the reads. Paired-end resequencing reads were mapped to the panda reference genome [19] with BWA Version: 0.7.12-r1039 [20] using the default parameters. Sequence Alignment/Map (SAM) format files were imported to Samtools Version: 0.1.18 [21] for sorting, merging, and converting mapping results into the BAM format. Duplicated reads were filtered with the Picard package (Version: 1.126). We then used Samtools again to remove duplicate, unmapped and discordantly mapping reads. Variants were called jointly across all samples using Samtools mpileup and bcftools call. Insertion/deletion variants were removed using bcftools view. Bcftools filter was used to remove SNPs meeting any of these criteria: located within 10bp of an indel, coverage across all samples of less than 20 reads or more than 800 reads, SNP spacing ≥10Kb, minor allele frequency (MAF) less than 10%, and QUAL value less than 20. This process called 6,993,226 SNPs. Given the abundance of the SNPs, we aimed at selecting SNPs with the highest quality by requiring each SNP to have no missing genotype for all forty-nine pandas and this requirement reduced the number of SNPs to 164,326. Of these SNPs, 150,025 SNPs passed the Hardy-Weinberg equilibrium test with p≥0.01 (SNPs with p<0.01 removed). To compare the estimates of genomic inbreeding and relatedness using a lower density SNP set, we selected 14,926 SNPs from the 150,025 SNPs with requirements of SNP spacing ≥ 150Kb, MAF ≥ 0.15 and passing HWE test with p≥0.05. The 150,025 SNPs will be referred to as the 150K SNP set, and the 14,926 SNPs the 15K SNP set.

### Genomic inbreeding and coancestry

The genomic inbreeding coefficient of an individual was calculated based on the individual’s own diagonal element in the genomic additive relationship matrix [13], and a coancestry coefficient between two individuals was calculated based on the two individuals’ off-diagonal element in the additive relationship matrix [11]. Genomic additive relationship matrix (**A**_g_) and genomic dominance relationship matrix (**D**_g_) were calculated using three definitions of genomic relationships. The three methods used in this study were Definitions I, IV and IVb implemented by GCORRMX program in the GVCBLUP program [11,22], where Definitions I used across-SNP standardization and Definition IV used within-SNP standardization [7], and Definition IVb [12,23] was similar to the within-SNP standardization of Definition IV except that the heterozygous genotype of each SNP was not used for diagonal elements of the additive relationship matrix. Genomic inbreeding coefficient of an individual (*f*) was calculated based on the diagonal element of the individual (A_ii_) in the **A**_g_ matrix, i.e.,

$$f=f_{i}={\text{A}}_{\text{ii}}-\text{1}.$$ (1)

The genomic inbreeding coefficients corresponding to Definitions I, IV and IVb will be denoted by *f*–I, *f*–IV and *f*–IVb respectively. The genomic mean coancestry coefficient within a habitat was defined as the average of all *f*_jk_ values between n pairs of individuals in the habitat, i.e.,

$$f_{\text{m}}=(\sum_{\text{i}=1}^{\text{n}}f_{\text{i}})/\text{n}$$ (2)

where subscript i denotes a j-k combination. Using the expectation that genomic additive relationship is twice the coancestry coefficient [11,24], genomic coancestry coefficient between individuals j and k (*f*_jk_) was calculated as:

$$f_{\text{jk}}=\;0.5{\text{A}}_{\text{jk}}=\text{genomic coancestry coefficient between individuals j and k}$$ (3)

where A_jk_ = genomic additive relationship between individuals j and k. Genomic dominance relationship or fraternity coefficient (*d*_jk_) between individuals j and k was calculated as the corresponding off-diagonal element in **D**_g_, i.e.,

$$d_{\text{jk}}={\text{D}}_{\text{jk}}.$$ (4)

To interpret the degree of severity of genomic inbreeding and coancestry coefficient as well as dominance relationships, we compare the genomic estimates with the pedigree expectations of typical relationships including parent-offspring, full-sibs and half-sibs [25], and with the expected increases in inbreeding coefficients of regular mating systems (S1 Fig).

### Additional measures of genomic relatedness and similarity

We estimated additional measures of genomic relatedness and similarity: probability of alleles identical by descent (IBD) to serve as a measure of genomic relatedness and a comparison with genomic conancestry coefficient, probability of alleles identical by state (IBS) as a measure of common alleles shared by two individuals, probability of SNP loci identical by genotype (IBG) as a measure of common genotypes shared by two individuals, and probability of non-shared genotypes between two genotypes (NSG) as a measure of opposing genotypes without common alleles (e.g., *AA* and *aa* genotypes) to exclude parent-offspring relationship between two individuals. In addition, multidimensional scaling (MDS) of the IBS matrix was calculated and plotted. The IBD, IBS and MDS measures were calculated using PLINK [26]. The MDS dimensions and PCA are highly correlated and the two methods would produce virtually the same plots for the same data. The purpose of MDS analysis was to study the difference in results between MDS and PCA due to difference in data utilization: the MDS analysis used filtered SNPs under highly restrictive conditions and used all forty-nine pandas, whereas the previous PCA analysis [1] used all SNPs from the whole-genome sequence data and used the thirty-four wild pandas only.

### Statistical tests of differences between wild panda populations

The differences between wild panda populations in genomic inbreeding coefficients, additive and dominance relationships, IBS, IBD, IBG and NSG were tested using the PROC GLM procedure of SAS [27]. The statistical model was y = μ + population + e, where y = the observation of any of the aforementioned variable, μ = common mean, population = effect of a wild panda population, and e = random residual. Four populations of Liangshan, Minshan, Qinling and Qionglai were included in the statistical model for genomic inbreeding coefficients, and Liangshn was omitted for testing all pairwise measures because Liangshan only had one pair of panda. The difference between two populations was estimated tested using the ‘Estimate’ option.

### SNP quality assessment and exclusion power

To assess the SNP quality after the SNP selection, we compared the observed and expected boundary values of IBS, IBG and NSG values. The expected IBS, IBG and NSG probabilities under HWE were derived as:

$${\text{IBS}}_{\text{E}}={\left({\text{p}}_{}^{\text{2}}+{\text{q}}_{}^{\text{2}}\right)}^{2}+2\text{pq}(1-\text{pq})$$ (5)

$${\text{IBG}}_{\text{E}}={\left({\text{p}}^{2}+\;{\text{q}}^{2}\right)}^{2}+\;2{\text{p}}^{2}{\text{q}}^{2}$$ (6)

$${\text{NSG}}_{\text{E}}=\;2{\text{p}}^{2}{\text{q}}^{2}$$ (7)

Where IBS_E_ = expected IBS probability of a SNP, IBS_E_ = expected IBG probability of a SNP, NSG_E_ = expected NSG probability of a SNP, P_11_ = genotypic frequency of *AA* genotype, P_22_ = genotypic frequency of *aa* genotype, p = frequency of allele *A* of the SNP, and q = frequency of allele *a* of the SNP. Under HWE, IBS and IBG reach their lower bounds whereas NSG reaches its upper bound at p = q = 0.5. With inbreeding, the expected NSG value is:

$${\text{NSG}}_{\text{Ef}}=\;2\left({\text{p}}^{2}+f\text{pq}\right)\left({\text{q}}^{2}+f\text{pq}\right)$$ (8)

where *f* = inbreeding coefficient. Eq 8 helps explain a non-intuitive observation in this study: a population with higher level of inbreeding also had a higher level of NSG. The expected values of IBS, IBG and NSG by Eqs 5–7 were compared with the observed values to identify observed outlier values to assess the quality of the 150K SNP set, and the result showed that only one outlier was observed, indicating excellent quality of the SNP set (S1 Text).

The NSG_E_ formula of Eq 7 assuming HWE is the same as the exclusion probability for a bi-allelic locus assuming genotypic availability of the child and the alleged parent in the absence of genotypic information from the other parent [28]. Therefore, the observed NSG is a measure of exclusion power of the SNP set for parentage testing. Assuming independent SNPs, the overall exclusion probability of n SNPs (Q) to exclude a false parent-offspring relationship between two randomly sampled pandas is: $\text{Q}=1-\Pi_{\text{i}=1}^{\text{n}}(1-{\text{Q}}_{\text{i}})$, where Q_i_ = NSG value for SNP i. Using the observed lower bound of NSG values between two pandas, the number of NSG SNPs among the 150,025 SNPs is 150,025 × 0.047 = 7051 SNPs. Using n = 7051 and Q_i_ = 0.017 (theoretical lower bound per SNP), the overall exclusion probability is: Q = 1–1.39(10)^−56^. Therefore, the expected exclusion power of the 150,025 SNPs to exclude a false parent-offspring relationship in pandas is virtually 100%. Even if the 150,025 SNPs only had 10% of independent SNPs, the overall exclusion would be Q = 0.9999974, still virtually 100%. Yet, these are the most conservative estimates assuming the smallest observed NSG value for any panda and the smallest expected NSG value per SNP.

## Results

Genomic inbreeding, additive and dominance relationships were estimated using two sets of SNP markers (the 150K and the 15K SNP sets) for forty-nine pandas including thirty-four wild pandas sampled from all six panda habitats, Minshan, Qionglai, Qinling, Liangshan, Daxiangling and Xiaoxiangling [1]. Four genomic similarity measures were also estimated using the 150K SNP set, including probability of alleles identical by descent (IBD) as a measure of genomic relatedness and a comparison with genomic conancestry coefficient, probability of alleles identical by state (IBS) as a measure of common alleles shared by two individuals, probability of SNP loci identical by genotype (IBG) as a measure of common genotypes shared by two individuals, and probability of non-shared genotypes between two genotypes (NSG) as a measure of opposing genotypes without common alleles (e.g., *AA* and *aa* genotypes) to exclude parent-offspring relationship between two individuals.

### Inbreeding levels in wild panda populations

**Fig 1:**
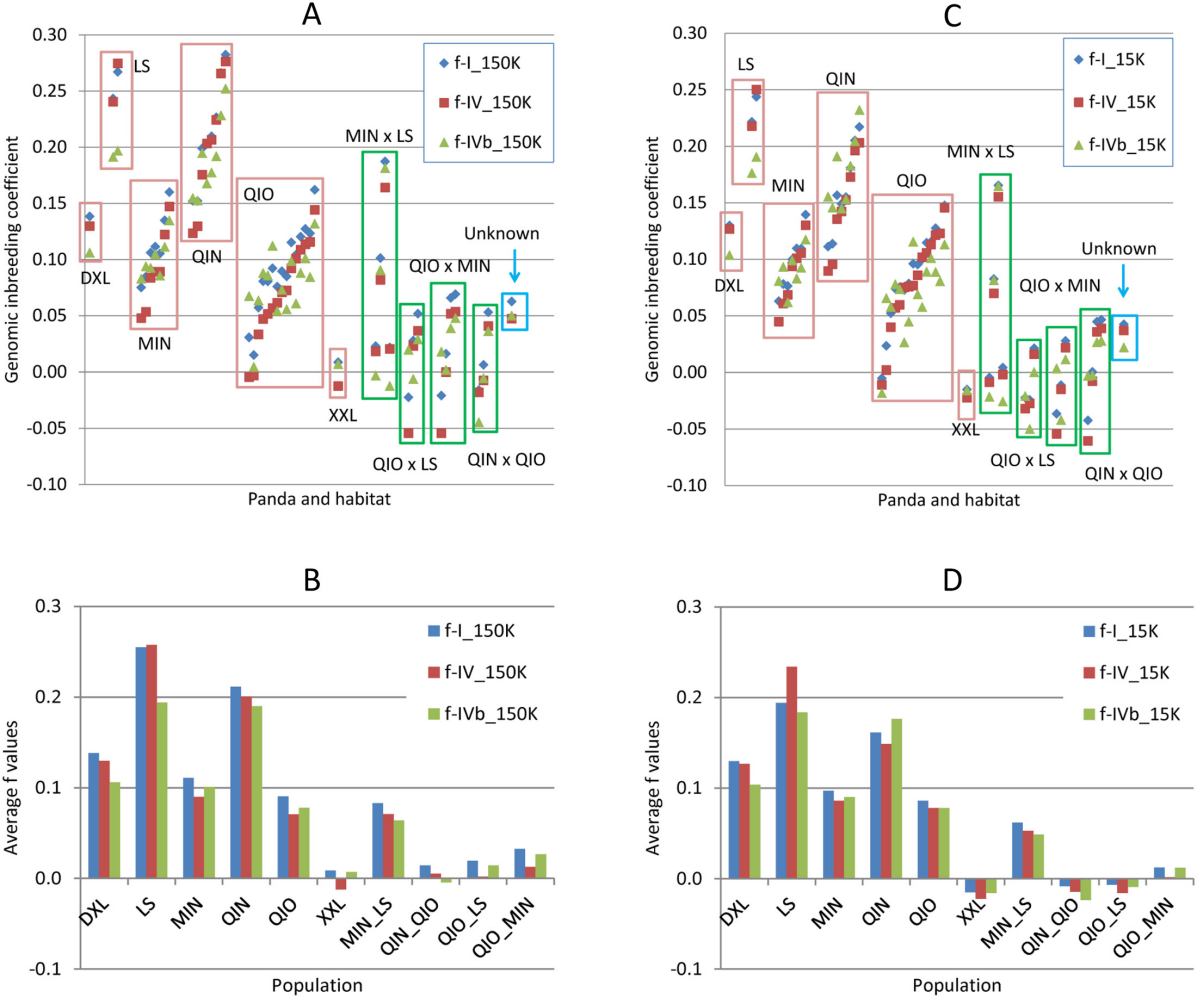
Genomic inbreeding coefficients of the 49 pandas including 34 wild pandas sampled from six habitats. **A:** Genomic inbreeding coefficients of all 49 pandas using the 150K SNP set. **B:** Average genomic inbreeding coefficients of all 48 pandas with known habitat origin using the 150K SNP set. **C:** Genomic inbreeding coefficients of all 49 pandas using the 15K SNP set. **D:** Average genomic inbreeding coefficients of all 48 pandas with known habitat origin using the 15K SNP set. The genomic inbreeding coefficient of each panda was calculated using three methods: *f*-I, *f*-IV and *f*-IVb based on Definitions I, IV and IVb of genomic additive relationships implemented by GVCBLUP [22]. Habitat abbreviations are: DXL = Daxiangling, LS = Liangshan, MIN = Minshan, QIN = Qinling, QIO = Qionglai, XXL = Xiaoxiangling.

Estimates of genomic inbreeding coefficient (*f*) of each panda by the three methods of Definitions I, IV and IVb [11,22] are to be denoted as *f*-I, *f*-IV and *f*-IVb respectively. Genomic inbreeding coefficients by these three methods mostly overlapped for individual pandas (Fig 1A and 1C). The averages of genomic inbreeding coefficients of all forty-nine pandas were the same for *f*-IV and *f*-IVb, and the average of genomic inbreeding coefficients of *f*-I was slightly higher than those of *f*-IV and *f*-IVb (Fig 1B and 1D). For individuals with high inbreeding coefficients, the 15K SNP set had lower estimates than those from the 150K SNP set for all three methods. Otherwise the 150K and 15K SNP sets had similar estimates (S1 Table).

The average genomic inbreeding coefficients ($\bar{f}$) calculated using the 150K and 15K SNPs showed that Qinling and Liangshan had the highest genomic inbreeding coefficients, with average inbreeding coefficient of *$\bar{f}$* = 0.148–0.201 for Qinling (N = 8) and $\bar{f}$ = 0.234–0.258 for Liangshan (N = 2), followed by Minshan ($\bar{f}$ = 0.086–0.090, N = 7), and Qionglai ($\bar{f}$ = 0.071–0.078, N = 15) (Table 1, Fig 1). Statistical tests showed that the genomic inbreeding coefficients of Qinling and Liangshan were significantly higher than those of Minshan and Qionglai (p<0.0001), and the difference in genomic inbreeding coefficients was insignificant between Qinling and Liangshan (p>0.1182) and between Minshan and Qionglai (p>0.3465) (Table 2**)**. Although Liangshan had significantly higher genomic inbreeding coefficients than Minshan and Qionglai, the interpretation of the Liangshan results requires caution because Liangshan only had two pandas in the sample. The lowest estimate of genomic inbreeding coefficient for the Qinling pandas (0.090) was above the average inbreeding levels of Minshan and Qionglai, indicating that the Qinling population likely had widespread inbreeding. The results of average inbreeding coefficients were consistent with the population sizes because the Qinling and Liangshan populations with high levels of inbreeding were smaller than the Minshan and Qionglai populations with relatively low levels of inbreeding. According to the Third National Panda survey, the numbers of pandas in Minshan, Qionglai, Qinling and Liangshan were 708, 437, 275 and 115 respectively [29] (Table 1), or, the Minshan and Qionglai populations were 1.6–6.2 times as large as the Qinling and Liangshan populations. The high inbreeding levels of the Qinling pandas were consistent with the habitat fragmentation and loss in Qinling [30–32]. Although Minshan and Qionglai had relatively low average genomic inbreeding coefficients, these two populations had pandas with substantial inbreeding coefficients: Minshan had two pandas (29%) with *f* ≥ 0.10 by *f*-IV and Qionglai had four such pandas (27%), and Minshan and Qionglai each had one panda with *f* ≥ 0.125 that is equivalent to the inbreeding level of half-sib mating (S1 Table). The Daxiangling panda had *f* = 0.127–0.130, and the Xiaoxiangling panda had no inbreeding (*f* = -0.022 to -0.012) (Table 1).

**Table 1:** Average genomic inbreeding coefficient ($\bar{f}$) by habitat^a^.

Habitat	N_w_^b^	N^c^	SNP set	Mean	SD	Min	Max
Daxiangling	29	1	150K	0.130	-	0.130	0.130
			15K	0.127	-	0.127	0.127
Liangshan	115	2	150K	0.258	0.024	0.241	0.275
			15K	0.234	0.023	0.218	0.250
Minshan	708	7	150K	0.090	0.024	0.048	0.147
			15K	0.086	0.030	0.045	0.130
Qinling	275	8	150K	0.201	0.056	0.123	0.276
			15K	0.148	0.042	0.090	0.203
Qionglai	437	15	150K	0.071	0.043	-0.004	0.144
			15K	0.078	0.044	-0.011	0.146
Xiaoxiangling	32	1	150K	-0.012	-	-0.012	-0.012
			15K	-0.022	-	-0.022	-0.022
Qionglai × Liangshan	-	3	150K	0.002	0.049	-0.022	0.052
			15K	-0.016	0.038	-0.054	0.022
Qionglai × Minshan	-	4	150K	0.013	0.043	-0.021	0.069
			15K	0.002	0.047	-0.060	0.039
Qionglai × Qinling	-	3	150K	0.005	0.035	-0.016	0.053
			15K	-0.014	0.026	-0.032	0.016
Minshan × Liangshan	-	4	150K	0.071	0.067	0.018	0.164
			15K	0.053	0.042	-0.008	0.155

^a^ Calculated from *f*-IV in S1 Table.

^b^ N_w_ = number of wild pandas according to The Third National Survey of Wild Panda Population [29][29].

^c^ N = number of pandas in this study.

**Table 2:** Significance test of differences in genomic inbreeding coefficients^a^ between habitats.

Comparison	Estimated difference	Standard error	t value	p value
Liangshan−Minshan	0.167	0.036	4.66	<.0001
Liangshan−Qinling	0.057	0.035	1.61	0.1182
Liangshan−Qionglai	0.187	0.034	5.55	<.0001
Minshan−Qinling	-0.110	0.023	-4.76	<.0001
Minshan−Qionglai	0.020	0.020	0.96	0.3465
Qinling−Qionglai	0.130	0.020	6.63	<.0001

^a^ Genomic inbreeding coefficients were calculated using the 150K SNP set and Definition IV.

Pandas from cross-breeding between habitats except two of the four Minshan × Liangshan crossbreds had the lowest genomic inbreeding coefficients ($\bar{f}$ = -0.016 to 0.005 by *f*-IV), consistent with the expectation that crossbred pandas between habitats without common ancestors should have ‘0’ inbreeding coefficients if different habitats are genetically independent as shown in the next section. Two Minshan × Liangshan crossbreds had genomic inbreeding coefficients *f* = 0.082–0.164 by *f*-IV using the 150K SNP set (estimates from *f*-I and *f*-IVb were slightly higher, S1 Table). The average inbreeding coefficients for all crossbreds including the two Minshan × Liangshan crossbreds with high *f* values was 0.025 using the 150K SNP set and was 0.009 using the 15K SNP set calculated from S1 Table, indicating negligible (0.009) to minor (0.025) potential bias in the genomic estimated inbreeding coefficients. Such negligible or minor potential bias should not change the interpretation that Qinling and Liangshan had high levels of inbreeding.

### Four genetically unrelated populations in the four largest habitats

**Fig 2:**
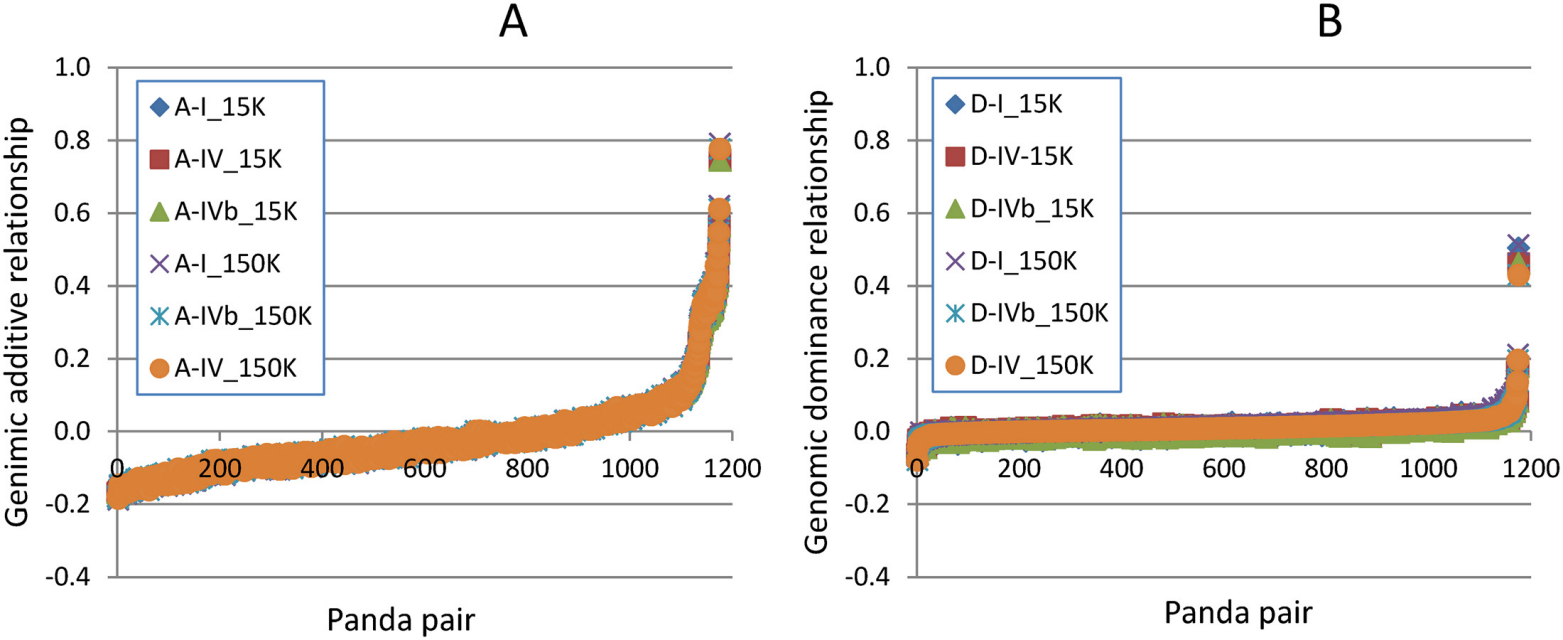
Estimates of genomic additive and dominance relationships using the 150K and 15K SNP sets and three estimation methods. **A:** Genomic additive relationships. **B:** Genomic dominance relationships. The three estimation methods were Definitions I, IV and IVb of additive and dominance relationships implemented by GVCBLUP [22].

Genomic coancestry coefficient and dominance relationship between each panda pair were estimated using the 150K and 15K SNP sets and the same three methods as for genomic inbreeding coefficients, and the six groups of estimates virtually completely overlapped except one pair of pandas that later will be shown to have several unexpected estimates (Fig 2). Therefore, results of coancestry coefficients and dominance relationships from any SNP set and any of the three methods were representative of all the six groups of estimates form the two SNP sets and three methods.

**Fig 3:**
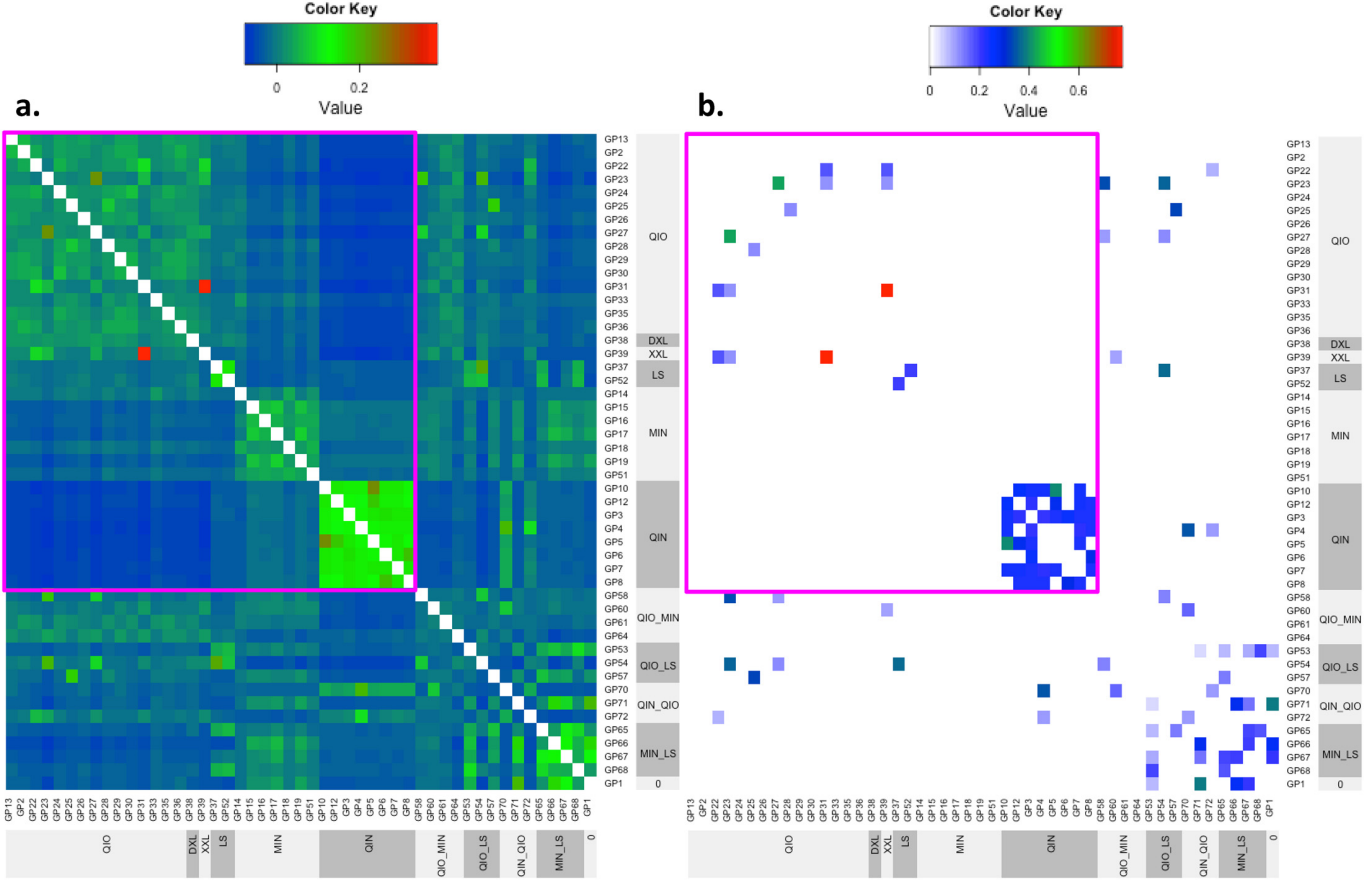
Global view of genomic coancestry coefficients and probability of alleles identical by descent (IBD). A: genomic coancestry coefficients. B: IBD probabilities. Both figures identified strong relatedness within Qinling and Liangshan, between the Xiaoxiangling panda and a Qionglai panda, and between some Qionglai pandas. Genomic coancestry coefficients clearly identified the Minshan population and identified relatedness between some Minshan pandas, whereas the IBD probabilities by PLINK did not identify the Minshan population as a result of finding no relatedness among the Minshan pandas. Both IBD and genomic coancestry found no relatedness or weak relatedness between the four largest habitats, Liangshan, Minshan, Qinling and Qionglai. These two figures identified Qinling and Liangshan to have high degrees of internal genomic relatedness, the four largest habitats (Liangshan, Minshan, Qinling and Qionglai) to be genetically unrelated to each other, and GP39 from Xiaoxiangling and GP31 from Qionglai to have the highest degree of genomic relatedness. Crossbreds between two habitats generally had visible genomic relatedness with their ancestral habitats, and the only panda with unknown origin had strong relationships with a Qinling × Qionglai crossbred, a Qionglai × Liangshan corossbred and two Minshan × Liangshan crossbreds.

**Fig 4:**
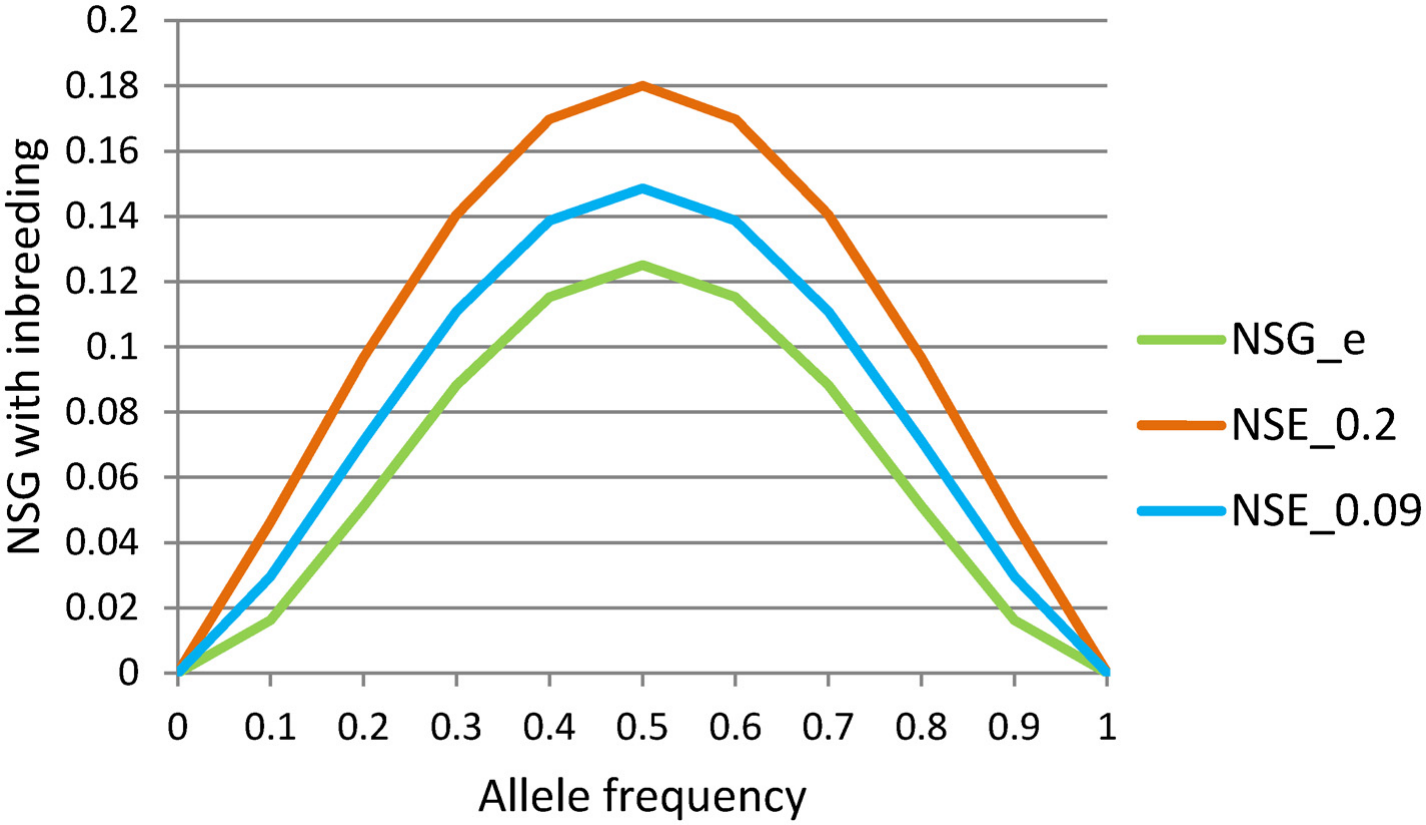
Probability of non-shared genotyped with inbreeding. NSG_e is the NSG value under HWE calculated using Eq 7, NSG_0.2 is the NSG value assuming *f* = 0.2 calculated using Eq 8, and NSG_0.09 is the NSG value assuming *f* = 0.09 calculated using Eq 8, with *f* = 0.2 being the inbreeding level of Qinling and *f* = 0.09 being the inbreeding level of Minshan. The figure shows that inbreeding increases NSG for all allele frequencies except the boundary values of ‘0’ and ‘1’.

Genomic coancestry coefficient clearly identified the wild panda populations in the four largest habitats of Minshan, Qionglai, Qinling and Liangshan to be genetically unrelated wild populations, whereas the estimated IBD probabilities by PLINK [26] did not identify the Minshan population as a result of not finding relatedness among the Minshan pandas (Fig 3). The IBD probabilities identified fifty-four panda pairs with nonzero IBD values ranging 0.046–0.775, and the corresponding genomic coancestry coefficients of those fifty-four pairs were in the range of 0.029–0.388 (S2 Table), showing strong mutual confirmation between IBD by PLINK and genomic coancestry coefficients for individual pairs with high levels of relatedness. The Qinling and Liangshan pandas had the highest genomic coancestry coefficients and similarity measures, whereas Minshan and Qionglai had the lowest genomic coancestry coefficients and similarity measures (Table 3), consistent with the high inbreeding coefficients in Qinling and Liangshan and the low inbreeding coefficients in Minshan and Qionglai. Statistical tests between the three largest habitats with more than one pair of pandas (Liangshan had only one pair and was excluded from the tests) showed that Qinling was significantly different from Minshan and Qionglai for all six measures of relatedness and similarity (p<0.0001), whereas the difference between Minshan and Qionglai was insignificant for all six measures of relatedness and similarity (p>0.1137) (Table 4), showing that the Qinling population was genetically different from the Minshan and Qionglai populations. The increased genomic coancestry coefficient, genomic dominance relationship, IBS, IBD and IBG over those in Minshan and Qionglai indicated increased genetic similarity among the Qinling pandas. The increased NSG value in Qinling and Liangshan was non-intuitive because NSG under HWE (Eq 7) is an increasing function of SNP heterozygosity, which should decrease with increased genetic similarity. Eq 8 shows that this non-intuitive increase in NSG values in Qinling and Liangshan in fact was expected due to higher inbreeding levels in Qinling and Liangshan. As shown in Fig 4, the increase in inbreeding coefficient increases NSG value for all allele frequencies except the boundary values of ‘0’ and ‘1’.

**Table 3:** Average genomic similarity measures of the four largest habitats^a^.

Habitat	# of pairs	*f*_m_	*d*_m_	IBD	IBS	IBG	NSG
Liangshan	1	0.190	0.098	0.201	0.760	0.582	0.096
Minshan	21	0.039	0.015	0.000	0.719	0.524	0.072
Qinling	28	0.186	0.060	0.166	0.770	0.604	0.090
Qionglai	105	0.028	0.009	0.008	0.718	0.517	0.071

^a^ Summarized from S3 Table. *f*_m_ = average of genomic coancestry coefficients, *d*_m_ = average of genomic dominance relationship, IBD = probability of identify by descent, IBS = probability of identity by state, IBG = probability of identity by genotype, and NSG = frequency of non-shared genotypes.

**Table 4:** Significance test of differences in relatedness and similarity between habitats.

Comparison	Relatedness or similarity	Estimated difference	Standard error	t value	p value
Qinling-Minshan	*f*_jk_	0.147	0.009	16.650	<.0001
	*d*_jk_	0.062	0.006	10.420	<.0001
	IBS	0.051	0.004	12.890	<.0001
	IBD	0.166	0.019	8.930	<.0001
	IBG	0.080	0.006	14.260	<.0001
	NSG	0.018	0.001	12.170	<.0001
Qinling-Qionglai	*f*_jk_	0.157	0.006	24.170	<.0001
	*d*_jk_	0.069	0.004	15.930	<.0001
	IBS	0.052	0.003	17.940	<.0001
	IBD	0.158	0.014	11.540	<.0001
	IBG	0.087	0.004	20.920	<.0001
	NSG	0.019	0.001	17.570	<.0001
Minshan-Qionglai	*f*_jk_	0.010	0.007	1.400	0.1634
	*d*_jk_	0.008	0.005	1.590	0.1137
	IBS	0.001	0.003	0.400	0.6906
	IBD	-0.008	0.015	-0.510	0.6103
	IBG	0.006	0.005	1.390	0.1666
	NSG	0.001	0.001	0.940	0.3494

*f*_jk_ = genomic coancestry coefficient between pandas j and k calculated by Definition IV, *d*_jk_ = genomic dominance relationship between pandas j and k calculated by Definition IV, IBS = probability of identity by state, IBD = probability of identity by descent, IBG = probability of identity by genotype, NSG = probability of non-shared genotypes.

The genetic differences between populations in different habitats can be further quantified by measures of relatedness and similarity between populations. The four largest populations of Qionglai, Minshan, Qinling and Liangshan had negative genomic mean coancestry coefficients (*f*_m_) calculated as the average of genomic coancestry coefficients between all pairs of individuals between two different habitats, ranging from *f*_m_ = −0.070 between Qinling and Qionglai to *f*_m_ = −0.010 between Liangshan and Qionglai (Table 5, S3 Table). Previously we interpreted negative estimates of genomic relationship as no correlation because negative genomic relationships were mostly observed in unrelated individuals, and we left open the question whether negative estimates of genomic relationship could be interpreted as negative genomic correlation [11]. With the interpretation of ‘genetic independence’ for zero or negative genomic coancestry coefficients, the four largest panda populations of Qionglai, Minshan, Qinling and Liangshan were genetically unrelated. With this result, crossbreds between habitats are expected to have no inbreeding.

**Table 5:** Average genomic similarity measures and distances between the four largest habitats^a^.

	Qionglai	Minshan	Qinling
Liangshan	*f*_m_ = -0.010	*f*_m_ = -0.032	*f*_m_ = -0.034
	IBS = 0.687	IBS = 0.674	IBS = 0.662
	IBG = 0.483	IBG = 0.469	IBG = 0.465
	NSG = 0.072	NSG = 0.068	NSG = 0.068
	km = 149	km = 265	km = 506
Qionglai		*f*_m_ = -0.024	*f*_m_ = -0.059
		IBS = 0.691	IBS = 0.660
		IBG = 0.483	IBG = 0.456
		NSG = 0.072	NSG = 0.060
		km = 151	km = 391
Minshan			*f*_m_ = -0.020
			IBS = 0.681
			IBG = 0.486
			NSG = 0.066
			km = 202

^a^ The measures of *f*_m_, IBS, IBG and NSG are summarized from S3 Table. *f*_m_ = average of genomic coancestry coefficients (*f*-IV), IBS = probability of identity by state, IBG = probability of identity by genotype, NSG = probability of non-shared genotypes, km = average distance between individuals calculated as the straight-line distance or ‘distance as the crow flies’ between two locations of DNA sample collection for the two pandas in different habitats using ‘FreeMapTools’, https://www.freemaptools.com/how-far-is-it-between.htm.

The genetic independence between the four largest populations shown by the *f*_m_ values in Table 5 and the patterns of genomic coancestry in Fig 3 was further confirmed by IBS, IBG, and NSG. The IBS probabilities distinguished the four largest panda populations as clearly as distinguished by genomic coancestry coefficients, and the IBG and NSG measures also showed the same patterns but not as clearly as those by genomic coancestry coefficients and IBS (S2 Fig). These results showed that the wild panda populations in the four largest habitats had their own unique genetic diversity.

To further evaluate the evidence from genomic relationships and similarity measures that the four largest populations of Liangshan, Minshan, Qinling and Qionglai were genetically unrelated, we analyzed multidimensional scaling (MDS) plots for all pairs of the first four MDS dimensions [26] for all forty-nine pandas (S3 Fig). The general patterns of the thirty-four wild pandas in the MDS plot of the first two dimensions were similar to those in the previously published PCA plot [1] except that the two Liangshan pandas shifted to one side of Qionglai, rather than straightly below Qionglai in the previous PCA plot. The two Liangshan pandas were at one extreme end of Dimension 3 of the MDS plots, with striking distances from the other populations, indicating that Liangshan pandas had their unique genome characteristics represented by Dimension 3. Qinling and Qionglai had the largest distance between them for Dimension 1 but virtually had no difference for Dimensions 2 and 3, and Minshan and Qionglai were always separated by similar distances on all six MDS plots. A difference between our MDS and previous PCA results was between the plot of MDS Dimensions 1 and 3 and the plot of PCA 1 and 3. The plot of MDS Dimensions 1 and 3 placed the two Liangshan pandas far from Minshan and Qionglai, whereas the plot of PCA 1 and 3 placed the two Liangshan pandas close to the Minshan pandas. It is known that the MDS dimensions and PCA are highly correlated and the two methods would produc virtually the same plots for the same data. Therefore, the difference between our MDS plots and the previous PCA plots was not due to the methodology difference between MDS and PCA. The MDS and PCA analyses had two differences in data utilization: 1) The MDS analysis used all forty-nine pandas whereas the PCA analysis used wild pandas only, and 2) The MDS analysis used filtered SNPs under highly restrictive conditions whereas PCA analysis used all SNPs from the whole-genome sequences. These differences in data utilization should be the apparent reason for the difference between the results of MDS and PCA. The MDS results added further evidence that Qionglai, Minshan, Qinling and Liangshan populations were four genetically unrelated panda populations.

The Daxiangling and Xiaoxiangling habitats (the two smallest habitats) each had one panda in this study. Results of these two pandas showed that the Daxiaingling and Qionglai populations either were the same population or shared common ancestors, whereas the Xiaoxiangling panda either migrated from Qionglai or had a problem of DNA sample mixing with a Qionglai panda.

The Daxiangling panda (GP38) had genomic relationship estimates consistent with those of a Qionglai panda. This panda had *f*_m_ = 0.022 with the fifteen Qionglai pandas, compared to *f*_m_ = 0.025 within Qionglai, and was unrelated to pandas in the other habitats (-0.069 ≤ *f*_m_ ≤ 0.007) (S3 Table). The highest coancestry coefficient (*f*_jk_) involving GP38 was 0.039 between GP38 and a Qionglai panda (GP27). Other than Qionglai pandas, the highest *f*_jk_ involving GP38 was *f*_jk_ = 0.004 between GP38 and a Minshan panda (GP14) (S2 Table). These results indicated that GP38 could be a member of Qionglai or had Qionglai ancestors. If this panda was representative of the Daxiangling population, Daxiangling and Qionglai populations either shared common ancestors or were the same population.

The Xiaoxiangling panda (GP39) and a Qionglai panda (GP31) had the highest genomic dominance relationship and coancestry coefficient: *d*_jk_ = 0.430 and *f*_jk_ = 0.388. The second highest values of any other panda pair were *d*_jk_ = 0.196 between GP23 and GP27 (two Qionglai pandas) and *f*_jk_ = 0.305 between GP5 and GP10 (two Qinling pandas) (S2 Table). The additive and dominance relationships of GP39 and GP31 were clearly much higher than those of any other panda pairs and were the outliers in Fig 2. The 0.775 estimate of IBD probability by PLINK between GP39 and GP31 was about twice as large as the genomic coancestry coefficient of 0.388, confirming the strong relationship between GP39 and GP31. These two pandas could be full-sibs for four reasons: 1) the *d*_jk_ = 0.430 and *f*_jk_ = 0.388 estimates exceeded the expected dominance relationships and coancestry coefficients of *d*_jk_ = *f*_jk_ = 0.25 for full-sibs or dizygotic twins assuming no pedigree inbreeding, 2) parent-offspring and half-sib relations were expected to have no dominance relationship (*d*_jk_ = 0) [25] and hence such relationships were excluded, 3) the observed 86.1% IBG between GP39 and GP31 (S2 Table) excluded the possibility of monozygotic twins or duplicate samples that are expected to have 100% IBG, and 4) parentage testing excluded the possibility of a parent-offspring relationship between GP39 and GP31 by 4.7% of the 150,025 SNPs (NSG value in S2 Table) with virtually 100% exclusion power, adding that the parentage testing excluded any parent-offspring relationship among the forty-nine pandas. Although evidence appeared to be strong in support of identifying GP39 and GP31 as full-sibs, the DNA samples for these two pandas could have been mixed together for two reasons: 1) these two pandas had extremely high estimates of genomic relatedness and similarity but each panda virtually had zero inbreeding coefficients, and 2) all the outliers and unexpected estimates of these two pandas could be explained by the assumption of mixed DNA samples of GP39 and GP31. Therefore, the results involving the Xiaoxiangling panda should be considered questionable.

Summarizing the analysis of wild pandas from all six habitats, the four largest panda populations in Minshan, Qionglai, Qinling and Liangshan were genetically unrelated with little genetic exchange between them. Qinling and Liangshan had highest internal levels of relatedness, and Minshan and Qionglai each had a panda with genomic relationships exceeding those expected from a half-sib mating system while on average had low levels of relatedness. The Daxiangling population either was part of the Qionglai population, or shared common ancestors with the Qionglai population, or had a panda migrated from Qionglai. The genetic status of Xiaoxiangling should be considered unknown.

### Genomic coancestry and geographical distance

**Fig 5:**
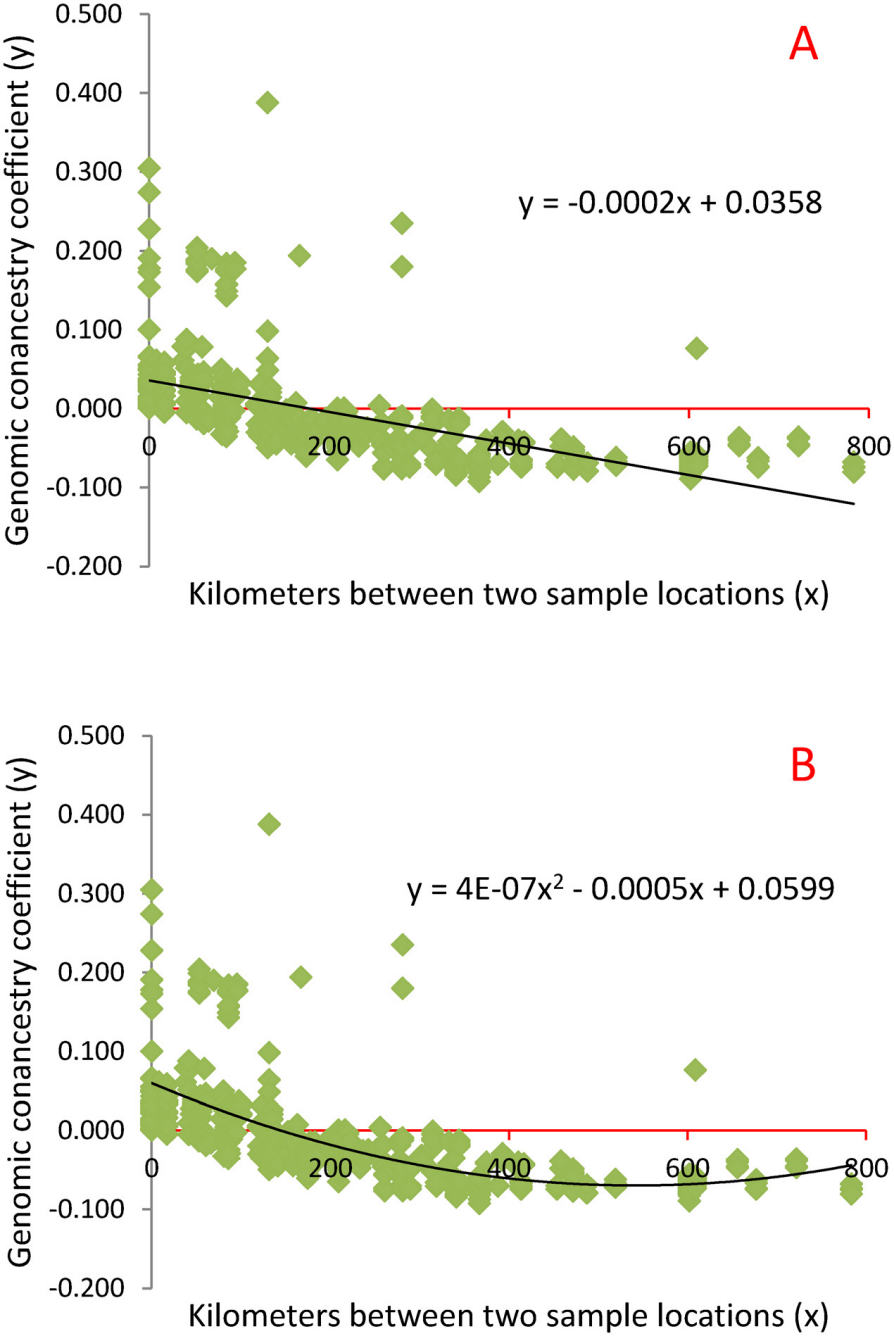
Regression of coancestry coefficients on the geographical distances between the locations of panda DNA sample collection. **A**: Linear regression model indicating a reduction of 0.02 in coancestry coefficient for every 100 kilometers increase in the geographical distance separating two pandas. **B**: Polynomial regression model indicating a reduction of 0.046 in coancestry coefficient for every 100 kilometers increase in the geographical distance separating two pandas.

The genomic mean coancestry coefficients (*f*_m_) between different habitats in Table 5 indicated an inverse relationship between genomic relatedness and the geographical distances separating the pandas. This relationship was true for all pairwise comparisons between the four largest habitats for the genomic mean coancestry coefficients (*f*_m_) and the average similarity measures of IBS, IBG and NSG between different habitats in Table 5, with the only exception of Liangshan-Qinling and Qionglai-Qinling. The geographical distance between Liangshan and Qinling was greater than between Qionglai and Qinling, but the genomic similarity between Liangshan and Qinling was slightly more than between Qionglai and Qinling. This exception could have been due to variations associated with the small number of pandas from Liangshan (two pandas). Regression analysis showed that for every 100 kilometers (km) reduction in the geographical distance separating two pandas, the coancestry coefficient between the two pandas would increase by 0.02 under a linear model (Fig 5) or increase by 0.046 under a polynomial model (Fig 5B). Under either model, pandas separated by 200 kilometers or more would share no IBD genes (*f*_jk_≤ 0), and this prediction was consistent with most of the actual observations. Although all similarity measures including IBS, IBG and NSG were significantly correlated with the geographical distances and with each other (p<0.0001, S4 Table), the IBS, IBG and NSG values did not have a strong regression on the geographical distances between each pair of pandas. The relationship between genomic coancestry coefficients and the geographical distances showed that geographical distances separating wild panda populations were effective in blocking gene flow between populations.

## Discussion

The results of genomic inbreeding and similarity measures provided estimates of actual levels of inbreeding and relatedness of wild pandas in their natural habitats and have important implications to panda conservation including captive breeding and reintroduction programs.

### High inbreeding level in Qinling and Liangshan adds to urgency for habitat protection

The Qinling and Liangshan pandas had surprisingly high levels of inbreeding and relatedness. The severity of the observed inbreeding levels could be assessed by comparing the observed with the expected inbreeding levels of regular mating systems. The average genomic inbreeding coefficients of $\bar{f}$ = 0.148–0.201 in Qinling was close to the expected inbreeding level of 1–2 generations of half-sib mating, and $\bar{f}$ = 0.234–0.258 of the Liangshan pair was at the level of one generation of full-sib mating that results in *f* = 0.25 (S1 Fig), noting that Liangshan had only two randomly sampled pandas. The lowest coancestry coefficient among the Qinling pandas (0.090) was above the average coancestry coefficients of Minshan and Qionglai, showing that inbreeding could be widespread among the Qinling pandas. The high levels of the observed genomic inbreeding in Qinling were consistent with the expectation that habitat loss and fragmentation in Qinling [30–32] would inevitably increase inbreeding. Such genomic evidence adds to the urgency to protect the Qinling habitat.

The high levels of inbreeding most likely were due to longtime accumulation of inbreeding rather than recent inbreeding between close relatives, as indicated by the low dominance relationships for most panda pairs within each habitat. Among full-sib, half-sib and parent-offspring relationships, only full-sibs are expected to have nonzero dominance relationships with the expected value of *d*_jk_ = 0.25. Another type of relationship with nonzero dominance relationship is double first cousin with expected dominance relationship of *d*_jk_ = 0.0625 [25]. The Qinling pandas had dominance relationships in the range of *d*_jk_ = 0.026–0.136 with an average dominance relationship of *d*_m_ = 0.060, and the two Liangshan pandas had a dominance relationship of *d*_jk_ = 0.098. Therefore, these observed dominance relationships were not those of full-sib, half-sib or parent-offspring relationship, and the wide range of dominance relationships of *d*_jk_ = 0.026–0.136 in Qinling indicated cumulative results of mating between remotely related pandas. A study using nineteen microsatellite markers also detected high levels of inbreeding among pandas of Qinling origin maintained at the Louguantai panda breeding center with 61.9% of the pandas (13 out of 21) having *f* ≥ 0.125 and 23.8% of the pandas having *f* ≥ 0.25 [3]. Since avoiding inbreeding has been a high priority in captive panda breeding [6], the high levels of inbreeding among the Louguantai pandas must have been carried into the captive breeding program from the wild Qinling founders prior to their introduction into the captive breeding program at Louguantai. Our results along with the microsatellite results indicated a longtime accumulation of inbreeding in Qinling and also indicated the risk of hidden inbreeding in breeding plans based on pedigree information.

### Relatively optimistic inbreeding status in Qionglai and Minshan

The inbreeding situation in the Qionglai and Minshan habitats was considerably more optimistic than in Qinling and Liangshan, because the inbreeding levels of $\bar{f}$ = 0.071–0.082 in Qionglai and Minshan were only 28–48% of those in Qinling and Liangshan. The two largest wild panda populations should continue to have low levels of inbreeding because a large number of breeding individuals increases the availability of unrelated mates and may slow down inbreeding increases even for the same half-sib mating (S1 Fig). However, hidden inbreeding could exist among Qionglai and Minshan founders due to the presence of genomic related pandas with substantial coancestry coefficients. The fact that these two largest habitats had the lowest inbreeding levels supports the expectation that large habitats reduce the risk of inbreeding.

### Unrelated habitats provide opportunity for habitat-controlled captive breeding

The result that the four largest habitats were genetically unrelated provides an opportunity to use habit-controlled breeding to avoid hidden inbreeding by using mates from different habitats because mates from different habitats are expected to share no IBD alleles. The habitat origin of a mating candidate can be determined from of the pedigree of the captive panda population [33]. This type of habitat-controlled mating would minimize the chance of inbreeding due to hidden relatedness between common founders of the mates except for the case of unknown panda migration from one habitat into another. The idea of habitat-controlled breeding is in agreement with the opinion encouraging exchange of breeding pandas between breeding centers to avoid inbreeding [34]. The opinion opposing the exchange between Louguantai pandas of Qinling origion and the Chengdu pandas of Sichuan origin concerned the loss of local adaptability that the Qinling pandas developed in adapting to their Qinling environments [3]. While noting this concern, we believe the genetic independence between the four largest habitats should be used to avoid inbreeding at least outside the Louguantai breeding center until genome-guided breeding becomes available for accurate estimation of genomic inbreeding and coancestry for all breeding pandas.

### Genome-guided breeding and conservation

Genome-guided breeding could reveal hidden inbreeding of a potential breeding pair by estimating the genomic coancestry coefficient of each potential mating pair. The threshold value of genomic coancestry coefficient to exclude a potential matting pairs from the breeding plan should be established by comparing observed genomic coancestry coefficients with those calculated from pandas with known relationships such as full-sibs, half-sibs, and parent-offspring. Genome-guided breeding should genotype SNPs for all available pandas particularly breeding pandas to provide complete understanding of panda relatedness. Genomic coancestry coefficient between each breeding pair can be used to predict the inbreeding level of the hypothetical offspring of this breeding pair because parental genomic coancestry coefficient was shown to have a high correlation with the offspring’s pedigree inbreeding coefficient [11]. The results of genomic inbreeding coefficients of the crossbreeding pandas indicated that habitat-controlled breeding could be an effective approach for avoiding hidden inbreeding, because the crossbreds virutally had no inbreeding ($\bar{f}$ = -0.016 to 0.005, Table 1) except two Minshan × Liangshan crossbreds that had inbreeding coefficients of 0.082 and 0.164 (S1 Table). Habitat-controlled breeding can serve as a transitional method towards genome-guided breeding until SNP data for all breeding pandas become available.

The implementation of genome-guided breeding within the framework of ‘genome-guided conservation’ is most ideal to fully utilize all available SNP information in captive and wild panda populations. A DNA bank for all captive and wild pandas is the foundation of genome-guided conservation, and such DNA bank should benefit panda conservation and research for many years to come. Accordingly, a database of panda sequences and SNP genotype data for all pandas should be established. The SNP data will serve multiple purposes for the captive and wild populations, including calculation of genomic inbreeding and coancestry, parentage and sex determination, updating pedigree using genomic coancestry for the captive population, genome-guided breeding in the captive population, construction of genomic pedigree for the wild populations, estimation of genetic diversity in the wild populations, and assessing the origin of random pandas. The knowledge of inbreeding and relatedness in wild pandas is helpful for assessing the genetic impact of reintroducing a captive-born panda into the wild (candidate panda). The relatedness between the candidate panda and different habitats can be assessed by analyzing the genomic coancestry coefficients between the candidate panda and wild pandas in different habitats. This analysis will provide genomic evidence for either preserving the genetic diversity by releasing the candidate panda into a habitat with similar genomes, or for reducing inbreeding by releasing the panda into a genetically unrelated habitat known to have a high level of inbreeding such as Qinling. The SNP database could provide rapid identification of the origin of random pandas such as rescued pandas and deceased pandas in the wild. An example of such random pandas would be the lone female panda that was killed in late 2014 in Yunnan province where no panda had been known to exit in modern times. Analysis of the SNP data of this lone female panda against the SNP database could identify its origin, and possibly its relatives if the SNP database were sufficiently complete in covering the captive and wild panda populations. Results of genomic coancestry and similarity in this study indicate that adding the sequence data of this lone random female panda to the sequence data of the forty-nine pandas in this study could yield evidence whether this panda was from any of the four largest populations, Minshan, Qionglai, Qinling and Liangshan. Ultimately, genome-guided breeding and conservation will provide complete information about each panda’s genome for breeding and reintroduction and will allow precision breeding and conservation.

### Comparison with previous estimates of inbreeding and relatedness using microsatellite markers

The estimates of panda inbreeding coefficients and relatedness using nineteen microsatellite markers [3] were the only such estimates using genetic markers prior to this study. The microsatellite study found high levels of inbreeding and relatedness for pandas of Qinling origin at the Louguantai breeding center, low inbreeding and relatedness for pandas of Sichuan origin (including Minshan, Qionglai, Liangshan, Daxiangling and Xiaoxiangling) from Chengdu, Wolong and Beijing breeding centers, and high levels of inbreeding and relatedness for some wild-born pandas although the origins of those wild-born pandas were not specified. TThe high levels of inbreeding among the Louguantai pandas of Qinling origin were consistent with our genome-wide SNP result that wild pandas in Qinling had high levels of inbreeding and relatedness. The low inbreeding coefficients of pandas from the other breeding centers were not directly comparable with results in our study because those captive-born pandas could have genetic contributions from different habitats resulting in null or low inbreeding. The finding of related wild-born pandas in the microsatellite study and the relatedness among some wild pandas in this study showed that hidden inbreeding due to related founders unobservable from the pedigree information likely existed in the captive population. A major difference between this microsatellite study and the whole-genome sequence study [1] as well as our current study using sequence-based SNP markers is the grouping of pandas of different habitat origin. The microsatellite study grouped the Louguantai pandas of Qinling origin and the Chengdu pandas of Sichuan origin as one group, whereas the two sequence-based studies classified the Qinling population and the Sichuan populations as genetically distinct populations. Differences in methodology also existed between the microsatellite study and our current study. Microsatellite markers typically are multi-allelic and such markers have not been used for estimating genomic inbreeding coefficient and conancestry coefficient, although a method to estimate genomic additive and dominance relationships using multi-allelic markers was recently developed [35]. Previous applications of genomic relationships all used genome-wide SNP markers, which are bi-allelic. The formulation for estimating inbreeding and coancestry using genetic markers [36] in the microsatellite study was different from that for estimating genomic relationships using multi-allelic markers [35]. The two studies also had difference in the number of markers used, nineteen microsatellite markers in the microsatellite study, and 15K-150K genome-wide SNPs in our current study.

### Consistency and specificity of genomic relationships in panda

Our cattle and swine studies showed that genomic additive and dominance relationships on average were remarkably close to the theoretical expectation of pedigree relationships, but within each type of relationship such as parent-offspring, full-sibs, half-sib or unrelated individuals, the actual estimates of a genomic relationship for different individuals may have large variations reflective of the genomic specificity of different individuals [11,24]. For this panda study, no panda with known pedigree relationship was available. Consequently, direct estimates of the consistency between the genomic and pedigree estimates of inbreeding and relationships were unavailable. However, the comparison of the genomic inbreeding coefficients of crossbreds with the genomic coancestry coefficients between habitats offered indication of potential consistency of the genomic relationships with pedigree relationships in panda. The slightly negative average genomic coancestry coefficients between habitats (Table 3) suggested that crossbreds between those habitats should have no inbreeding, and this expectation was close to what was observed from the crossbreds between those habitats. The fourteen crossbreds including the two Minshan × Liangshan crossbreds with high genomic inbreeding coefficients of 0.082 and 0.164 had low average genomic inbreeding coefficient of $\bar{f}$ = 0.025 by *f*-IV using the 150K SNP set (calculated from S1 Table) or negligible $\bar{f}$ = 0.009 by *f*-IV using the 15K SNP set. Therefore, the results of the crossbreds offered encouraging indication about potential consistency between genomic and pedigree relationships in panda.

### SNP abundance of whole-genome sequence data compensates for small sample size

Genomic additive and dominance relationships are calculated from SNP allele frequencies. Therefore, the accuracies of the estimated SNP allele frequencies affect the accuracies of genomic relationships. For a small sample size, missing SNP genotypes make the already small sample size even smaller. The SNP abundance of whole-genome sequence data provides opportunity to select SNPs without missing SNP genotypes in a small sample. With the 6,993,226 SNPs we called from the whole-genome sequence data of forty-nine pandas, we could afford requiring no missing SNP data for any SNP on any of the forty-nine pandas. This requirement reduced the number of eligible SNPs from nearly seven million to 164,330 that was further reduced to 150,025 by the HWE test with p≥0.01. The strict requirement of no missing SNP data allowed every SNP to have the maximum number of observations and hence provided a compensation for the small sample size. As a quality checking procedure, we examined whether any pair of pandas had outlier values for IBS, IBG and NSG by comparing the observed with the expected values (Methods), and found only two outliers involving the same pair of individuals among the 3528 observed values (S1 Text), indicating an excellent SNP quality and an acceptable sample size. A comparison of several MAF levels showed that higher MAF had higher correlation between the genomic and pedigree relationships [14], and a comparison of MAF levels for genomic selection showed increased prediction accuracy with higher MAF [37]. In contrast to a SNP chip with a fixed number of SNPs, whole-genome sequence data has the advantage to offer high quality SNPs to fully utilize small samples.

## Conclusion

The approach of genomic relationships allows the estimation of inbreeding and coancestry of wild pandas without pedigree information. The high levels of genomic inbreeding and relatedness in Qinling known to have habitat loss and fragmentation confirms the anticipation that habitat loss and fragmentation would increases inbreeding, and the relatively low inbreeding and relatedness in the two largest habitats of Minshan and Qionglai supports the hypothesis of reduced inbreeding in large habitats. Genetic differentiation between the four largest habitats was related to their geographical distances, and the four largest habitats were genetically independent and had their own genetic identity. These results lead to the recommendation of habitat-controlled breeding and genome-guided breeding and conservation for the giant panda.

## Supporting Information

S1 Fig Expected increases in inbreeding coefficients of regular mating systems. **A**: Full-sib mating. **B**: Backcross or parent-offspring mating. **C**: Half-sib mating with three sibs. **D**: Half-sib mating with four sibs showing slower inbreeding increases than half-sib mating with three sibs.
(PDF)

S2 Fig Global view of additional measures of genomic relatedness and similarity. **A**: Probability of alleles identical by state (IBS) as a measure of common alleles shared by a pair of individuals. **B**: Probability of SNP loci identical by genotype (IBG) as a measure of common genotypes shared by a pair of individuals. **C**: Probability of non-shared genotypes (NSG) as a measure of two genotypes of a pair of individuals without at least one common allele. **D**: Dominance relationship or fraternity coefficient (*d*_jk_) that is particularly useful for identifying full-sibs. These figures provided additional evidence that Qinling and Liangshan to have high degrees of genomic relatedness and similarity, and the four largest habitats (Minshan, Qionglai, Qinling and Liangshan) to be genetically unrelated. Crossbreds between two habitats generally had visible genomic relatedness with their ancestral habitats for all similarity measures except dominance relationships.
(PDF)

S3 Fig Multidimensional scaling (MDS) plot for each pair of the first four dimensions of the SNP IBS distances. C1-C4 are the four MDS dimensions calculated by PLINK [26]. IBS = probability of alleles identical by state.
(PDF)

S1 Table Genomic inbreeding coefficients of 49 pandas including 34 wild pandas calculated using the 150K and 15K SNP sets. (PDF)

S2 Table Estimated genomic relatedness and similarity measures between each pair of pandas within and between habitats using the 150K SNP markers. (PDF)

S3 Table Mean, standard deviation and range of pairwise genomic similarity measures within and between habitats calculated from S2 Table. (PDF)

S4 Table Correlations between geographical distances and measures of genomic relatedness and similarity. (PDF)

S1 Text Expected and observed values of probability of identify by state (IBS), probability of identity by genotype (IBG) and probability of non-shared genotypes (NSG) for identifying outliers of observed values. (PDF)
