# Supplementary material for: Genomic Inbreeding and Relatedness in Wild Panda Populations
Source: PLoS One. 2016 Aug 5;11(8):e0160496. doi: 10.1371/journal.pone.0160496 (PMC4975500; doi:10.1371/journal.pone.0160496)
Supplement: S1 Fig — A: Full-sib mating. B: Backcross or parent-offspring mating. C: Half-sib mating with three sibs. D: Half-sib mating with four sibs showing slower inbreeding increases than half-sib mating with three sibs. (PDF) [file pone.0160496.s001.pdf]

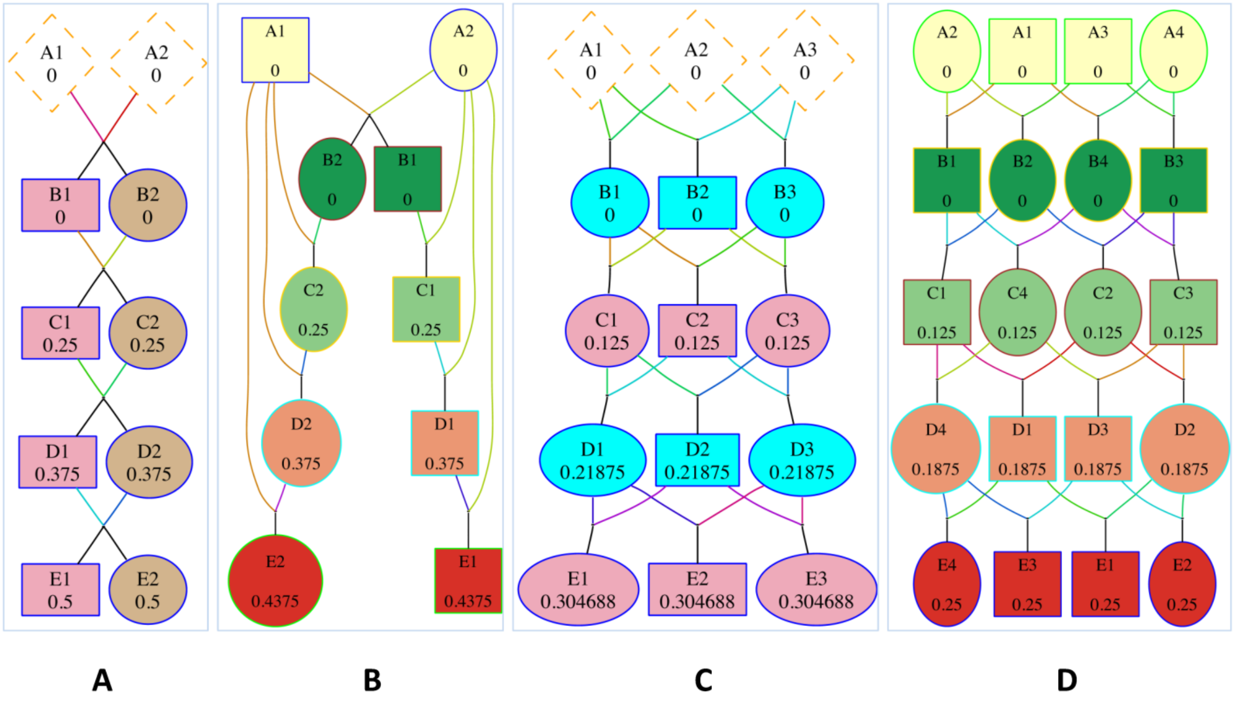

S1 Fig. Expected increases in inbreeding coefficients of regular mating systems. **A**: Full-sib mating. **B**: Backcross or parent-offspring mating. **C**: Half-sib mating with three sibs. **D**: Half-sib mating with four sibs showing slower inbreeding increases than half-sib mating with three sibs.
