# Supplementary material for: Genomic Inbreeding and Relatedness in Wild Panda Populations
Source: PLoS One. 2016 Aug 5;11(8):e0160496. doi: 10.1371/journal.pone.0160496 (PMC4975500; doi:10.1371/journal.pone.0160496)
Supplement: S2 Fig — A: Probability of alleles identical by state (IBS) as a measure of common alleles shared by a pair of individuals. B: Probability of SNP loci identical by genotype (IBG) as a measure of common genotypes shared by a pair of individuals. C: Probability of non-shared genotypes (NSG) as a measure of two genotypes of a pair of individuals without at least one common allele. D: Dominance relationship or fraternity coefficient (djk) that is particularly useful for identifying full-sibs. These figures provided additional evidence that Qinling and Liangshan to have high degrees of genomic relatedness and similarity, and the four largest habitats (Minshan, Qionglai, Qinling and Liangshan) to be genetically unrelated. Crossbreds between two habitats generally had visible genomic relatedness with their ancestral habitats for all similarity measures except dominance relationships. (PDF) [file pone.0160496.s002.pdf]

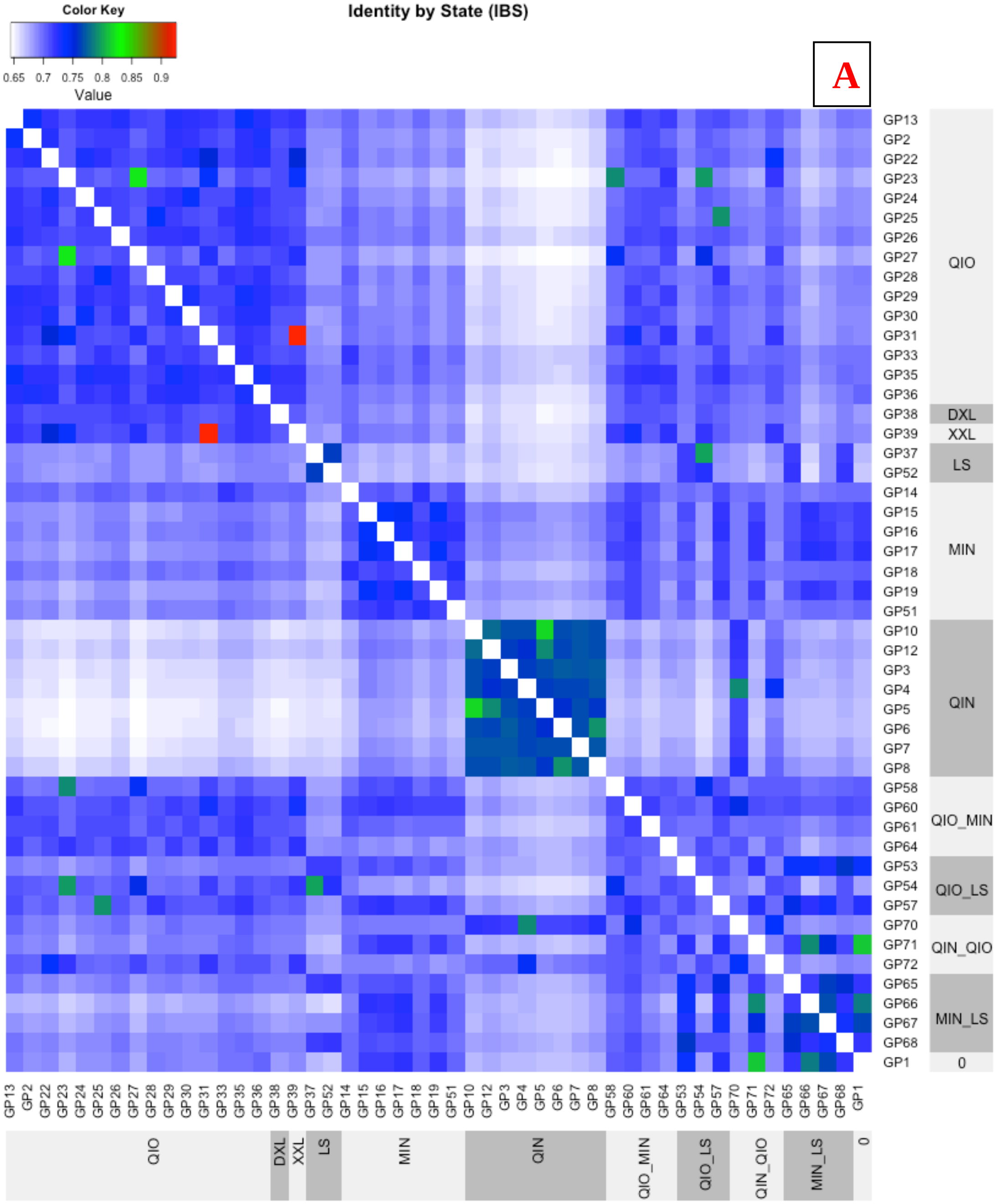

Color Key
0.65 0.7 0.75 0.8 0.85 0.9
Value
Identity by State (IBS)
A
GP13 GP2 GP22 GP23 GP24 GP25 GP26 GP27 GP28 GP29 GP30 GP31 GP33 GP35 GP36 GP38 GP39 GP37 GP52 GP14 GP15 GP16 GP17 GP18 GP19 GP51 GP10 GP12 GP3 GP4 GP5 GP6 GP7 GP8 GP58 GP60 GP61 GP64 GP53 GP54 GP57 GP70 GP71 GP72 GP65 GP66 GP67 GP68 GP1
QIO
DXL
XXL
LS
MIN
QIN
QIO_MIN
QIO_LS
QIN_QIO
MIN_LS
0

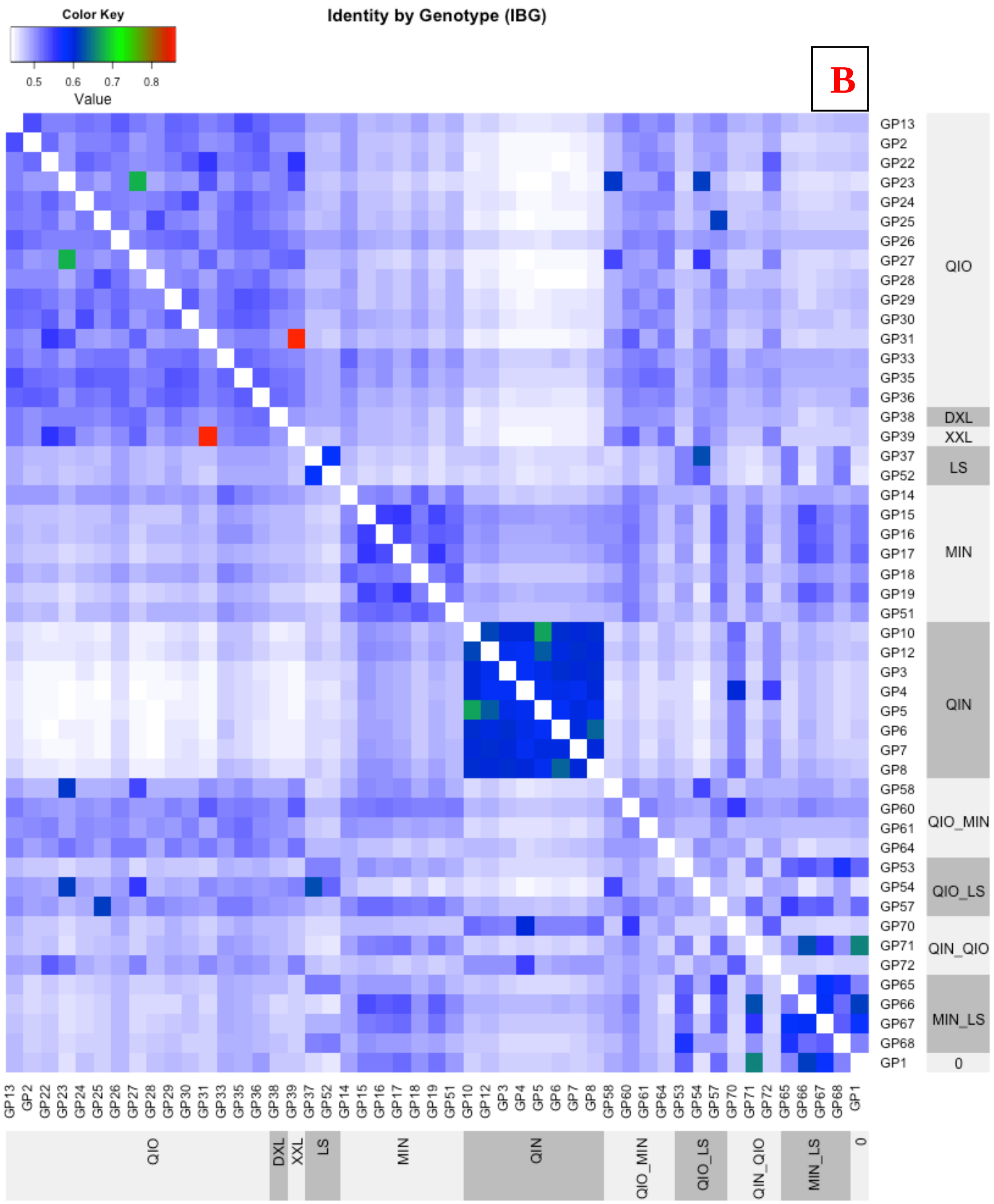
Color Key
0.5 0.6 0.7 0.8
Value
Identity by Genotype (IBG)
B
GP13
GP2
GP22
GP23
GP24
GP25
GP26
GP27
GP28
GP29
GP30
GP31
GP33
GP35
GP36
GP38
GP39
GP37
GP52
GP14
GP15
GP16
GP17
GP18
GP19
GP51
GP10
GP12
GP3
GP4
GP5
GP6
GP7
GP8
GP58
GP60
GP61
GP64
GP53
GP54
GP57
GP70
GP71
GP72
GP65
GP66
GP67
GP68
GP1
QIO
DXL
XXL
LS
MIN
QIN
QIO_MIN
QIO_LS
QIN_QIO
MIN_LS
0

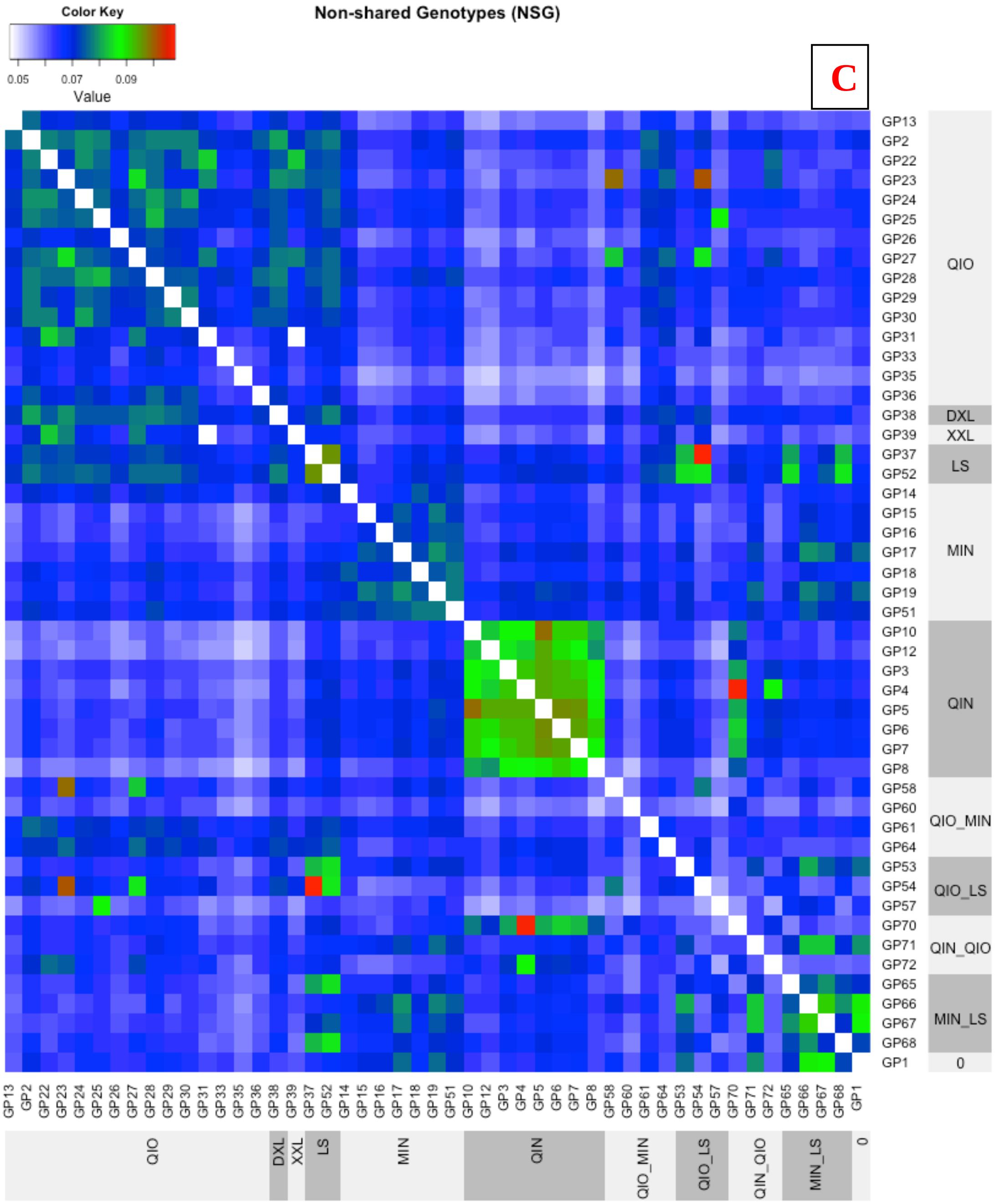
Color Key
0.05
0.07
0.09
Value
Non-shared Genotypes (NSG)
C
GP13 GP2 GP22 GP23 GP24 GP25 GP26 GP27 GP28 GP29 GP30 GP31 GP33 GP35 GP36 GP38 GP39 GP37 GP52 GP14 GP15 GP16 GP17 GP18 GP19 GP51 GP10 GP12 GP3 GP4 GP5 GP6 GP7 GP8 GP58 GP60 GP61 GP64 GP53 GP54 GP57 GP70 GP71 GP72 GP65 GP66 GP67 GP68 GP1
QIO
DXL
XXL
LS
MIN
QIN
QIO_MIN
QIO_LS
QIN_QIO
MIN_LS
0

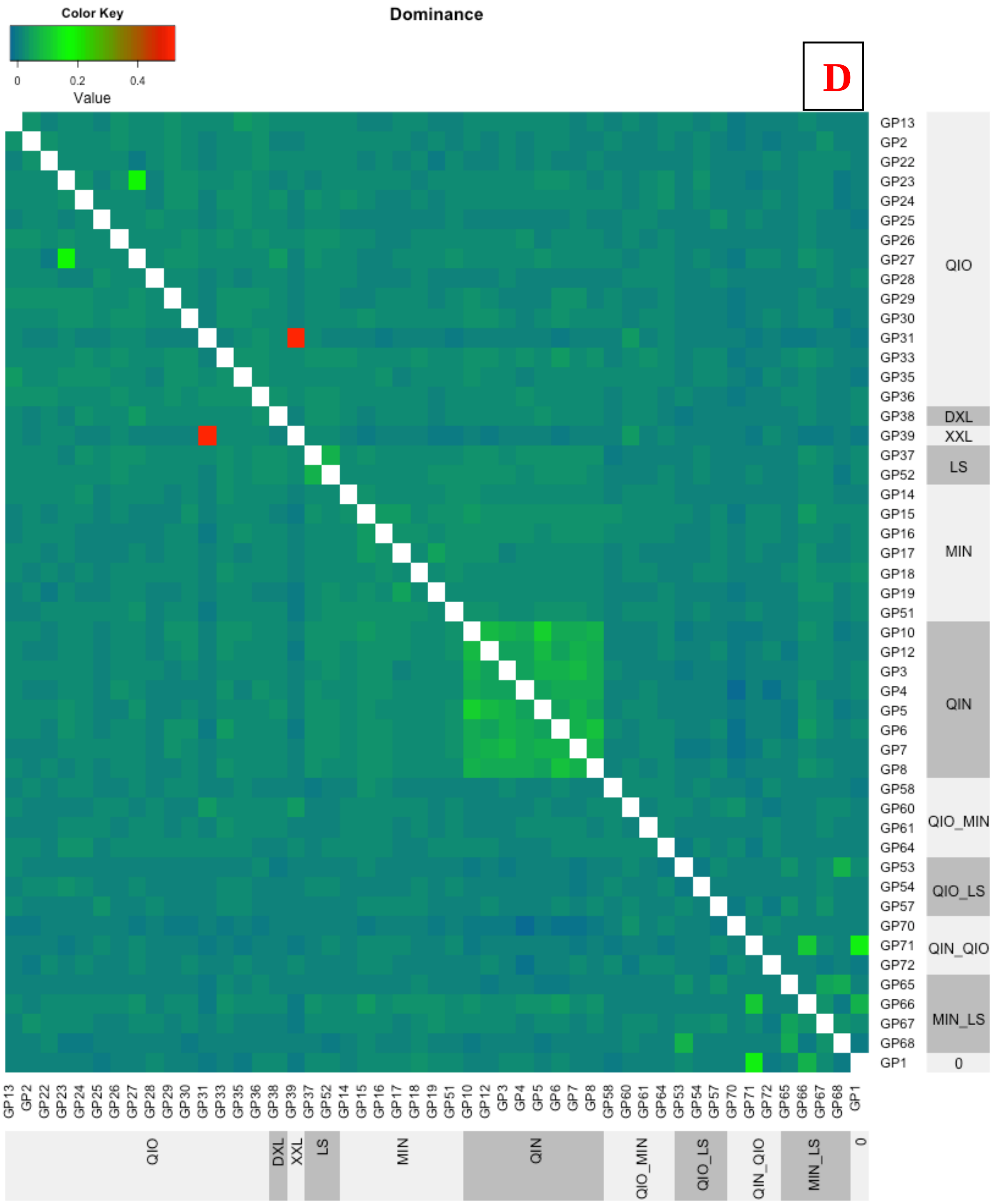

S2 Fig. Global view of additional measures of genomic relatedness and similarity. **A**: Probability of identical by state (IBS) as a measure of common alleles shared by a pair of individuals. **B**: Frequency of identical by genotype (IBG) as a measure of common genotypes shared by a pair of individuals. **C**: Frequency of non-shared genotypes (NSG) as a measure of two genotypes of a pair of individuals without at least one common allele. **D**: Dominance relationship or fraternity coefficient (d_{jk}) that is particularly useful for identifying full-sibs. These figures provided additional evidence that Qinling and Lianshan to have high degrees of genomic relatedness and similarity, and the four largest habitats (Minshan, Qionglai, Qinling and Liangshan) to be genetically unrelated. Crossbreds between two habitats generally had visible genomic relatedness with their ancestral habitats for all similarity measures except dominance relationships.
