# Supplementary material for: Genomic Inbreeding and Relatedness in Wild Panda Populations
Source: PLoS One. 2016 Aug 5;11(8):e0160496. doi: 10.1371/journal.pone.0160496 (PMC4975500; doi:10.1371/journal.pone.0160496)
Supplement: S3 Fig — C1-C4 are the four MDS dimensions calculated by PLINK [26]. IBS = probability of alleles identical by state. (PDF) [file pone.0160496.s003.pdf]

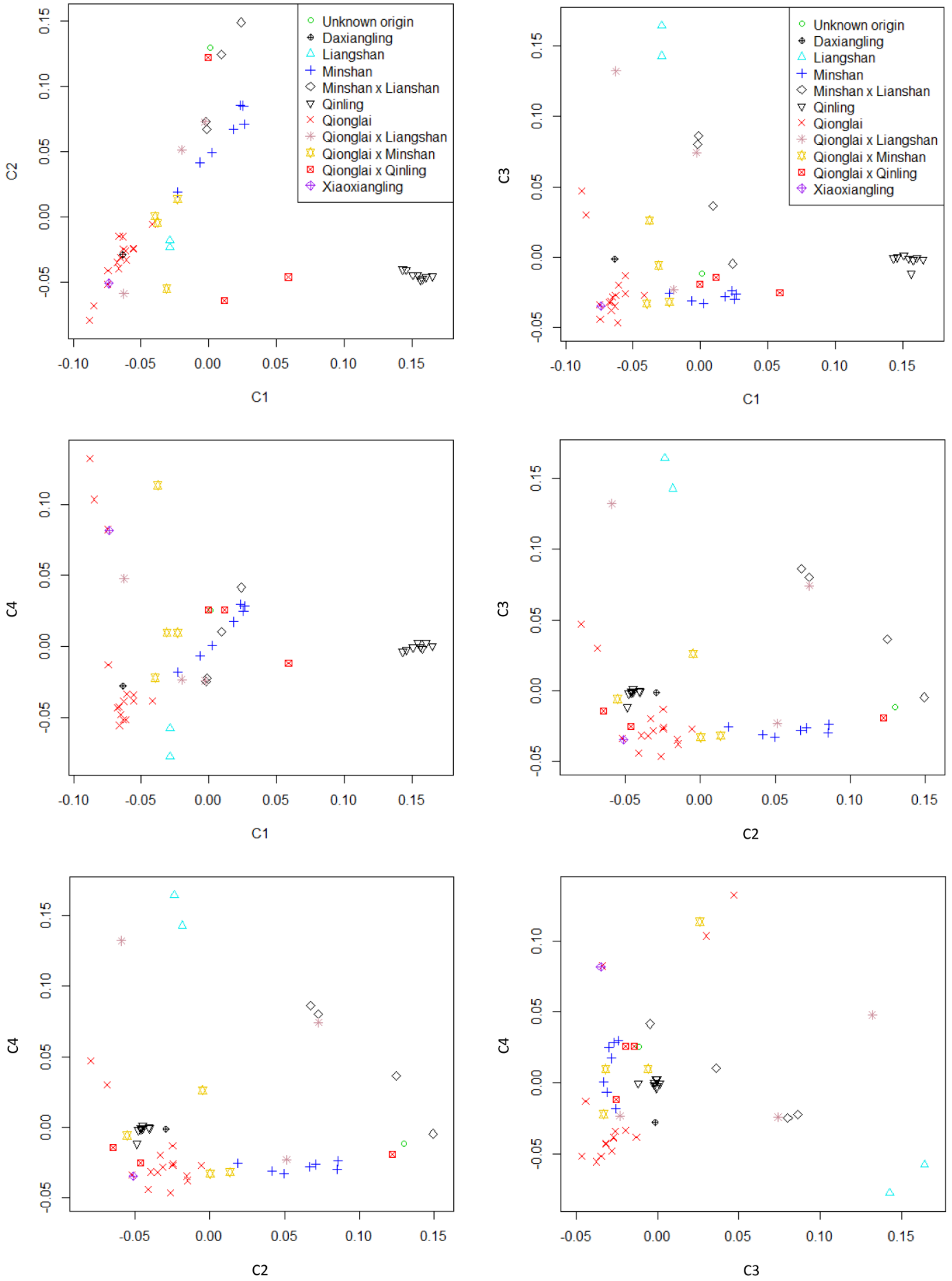

S3 Fig. **Multidimensional scaling (MDS) plot for each pair of the first four dimensions of the SNP identity by state (IBS) distances.** C1-C4 are the four MDS dimensions calculated by PLINK.
