# Supplementary material for: Genomic Inbreeding and Relatedness in Wild Panda Populations
Source: PLoS One. 2016 Aug 5;11(8):e0160496. doi: 10.1371/journal.pone.0160496 (PMC4975500; doi:10.1371/journal.pone.0160496)
Supplement: S1 Table — (PDF) [file pone.0160496.s004.pdf]

S1 Table. Genomic inbreeding coefficients of 49 pandas including 34 wild pandas calculated using the 150K and 15K SNP sets.

panda	habitat	150K SNP set			15K SNP set		
		f-I	*f*-IV	*f*-IVb	*f*-I_	*f*-IV	*f*-IVb
GP38	DXL	0.138	0.130	0.106	0.130	0.127	0.104
GP52	LS	0.267	0.275	0.197	0.244	0.250	0.191
GP37	LS	0.243	0.241	0.191	0.222	0.218	0.176
GP51	MIN	0.160	0.147	0.135	0.140	0.130	0.117
GP19	MIN	0.135	0.122	0.111	0.109	0.106	0.092
GP17	MIN	0.106	0.084	0.093	0.110	0.101	0.083
GP14	MIN	0.112	0.089	0.105	0.100	0.094	0.099
GP18	MIN	0.105	0.089	0.086	0.076	0.069	0.062
GP16	MIN	0.075	0.048	0.083	0.078	0.061	0.093
GP15	MIN	0.085	0.053	0.094	0.063	0.045	0.081
GP6	QIN	0.282	0.276	0.252	0.217	0.203	0.232
GP5	QIN	0.266	0.266	0.228	0.205	0.196	0.205
GP7	QIN	0.227	0.224	0.192	0.181	0.173	0.183
GP4	QIN	0.210	0.207	0.177	0.155	0.153	0.153
GP3	QIN	0.203	0.203	0.168	0.149	0.142	0.145
GP10	QIN	0.199	0.176	0.195	0.157	0.136	0.191
GP8	QIN	0.152	0.130	0.153	0.114	0.096	0.146
GP12	QIN	0.152	0.123	0.155	0.111	0.090	0.155
GP27	QIO	0.162	0.144	0.132	0.148	0.146	0.113
GP2	QIO	0.123	0.116	0.084	0.123	0.123	0.081
GP24	QIO	0.120	0.109	0.088	0.128	0.121	0.089
GP23	QIO	0.127	0.114	0.101	0.116	0.113	0.101
GP30	QIO	0.115	0.092	0.099	0.115	0.106	0.089
GP25	QIO	0.085	0.073	0.056	0.100	0.102	0.058
GP29	QIO	0.090	0.071	0.073	0.095	0.086	0.069
GP33	QIO	0.092	0.057	0.112	0.096	0.077	0.116
GP22	QIO	0.076	0.062	0.054	0.079	0.076	0.045
GP28	QIO	0.104	0.101	0.061	0.073	0.075	0.027
GP36	QIO	0.081	0.047	0.088	0.075	0.060	0.074
GP26	QIO	0.081	0.052	0.086	0.074	0.057	0.078
GP13	QIO	0.057	0.033	0.063	0.052	0.040	0.058
GP35	QIO	0.031	-0.004	0.067	0.024	0.002	0.066
GP31	QIO	0.015	-0.003	0.004	-0.005	-0.011	-0.018
GP39	XXL	0.009	-0.012	0.007	-0.015	-0.022	-0.016
GP66	MIN × LS	0.187	0.164	0.181	0.165	0.155	0.165
GP67	MIN × LS	0.101	0.082	0.091	0.083	0.070	0.081
GP65	MIN × LS	0.023	0.018	-0.003	-0.004	-0.009	-0.021
GP68	MIN × LS	0.022	0.021	-0.012	0.004	-0.002	-0.026

GP71	QIN × QIO	0.053	0.041	0.036	0.021	0.016	0.000
GP70	QIN × QIO	-0.016	-0.018	-0.045	-0.024	-0.027	-0.050
GP72	QIN × QIO	0.006	-0.007	-0.005	-0.022	-0.032	-0.021
GP54	QIO × LS	0.052	0.037	0.029	0.028	0.022	0.011
GP53	QIO × LS	0.028	0.023	-0.006	-0.011	-0.015	-0.042
GP57	QIO × LS	-0.022	-0.054	0.020	-0.037	-0.054	0.003
GP61	QIO × MIN	0.069	0.054	0.048	0.047	0.039	0.028
GP64	QIO × MIN	0.066	0.052	0.039	0.045	0.036	0.027
GP58	QIO × MIN	0.016	0.000	0.002	0.000	-0.008	-0.002
GP60	QIO × MIN	-0.021	-0.054	0.018	-0.042	-0.060	-0.003
GP1	Unknown	0.063	0.047	0.050	0.043	0.037	0.022

DXL = Daxiangling, LS = Liangshan, MIN = Minshan, QIN = Qinling, XXL = Xiaoxiangling.
f-I, *f*-IV and *f*-IVb are the genomic inbreeding coefficient calculated from diagonal elements of Definitions I, IV and IVb of genomic additive relationship matrix implemented by GVCBLUP.
