# Supplementary material for: Genomic Inbreeding and Relatedness in Wild Panda Populations
Source: PLoS One. 2016 Aug 5;11(8):e0160496. doi: 10.1371/journal.pone.0160496 (PMC4975500; doi:10.1371/journal.pone.0160496)
Supplement: S2 Table — (PDF) [file pone.0160496.s005.pdf]

S2 Table. Estimated genomic relatedness and similarity measures between each pair of pandas within and between habitats using the 150K SNP markers.

panda1	panda2	habitat1	habitat2	f_{jk}-IV	d_{jk}-IV	IBD	IBS	IBG	NSG
GP38	GP52	DXL	LS	0.015	0.027	0.000	0.692	0.490	0.078
GP37	GP38	LS	DXL	-0.002	0.030	0.000	0.689	0.488	0.073
GP14	GP38	MIN	DXL	0.004	0.008	0.000	0.701	0.498	0.071
GP18	GP38	MIN	DXL	-0.015	0.011	0.000	0.693	0.486	0.069
GP38	GP51	DXL	MIN	-0.025	0.017	0.000	0.685	0.479	0.070
GP16	GP38	MIN	DXL	-0.032	0.017	0.000	0.688	0.481	0.062
GP15	GP38	MIN	DXL	-0.033	0.011	0.000	0.686	0.479	0.064
GP17	GP38	MIN	DXL	-0.038	0.016	0.000	0.683	0.475	0.066
GP19	GP38	MIN	DXL	-0.038	0.009	0.000	0.679	0.469	0.068
GP12	GP38	QIN	DXL	-0.062	0.020	0.000	0.665	0.461	0.060
GP38	GP8	DXL	QIN	-0.064	0.017	0.000	0.663	0.459	0.060
GP3	GP38	QIN	DXL	-0.066	0.023	0.000	0.658	0.453	0.063
GP38	GP6	DXL	QIN	-0.070	0.030	0.000	0.652	0.451	0.064
GP38	GP7	DXL	QIN	-0.070	0.027	0.000	0.655	0.451	0.064
GP38	GP4	DXL	QIN	-0.071	0.020	0.000	0.657	0.450	0.063
GP10	GP38	QIN	DXL	-0.073	0.021	0.000	0.656	0.453	0.060
GP38	GP5	DXL	QIN	-0.074	0.033	0.000	0.650	0.450	0.063
GP27	GP38	QIO	DXL	0.039	0.024	0.000	0.715	0.522	0.077
GP29	GP38	QIO	DXL	0.036	0.008	0.000	0.717	0.518	0.076
GP26	GP38	QIO	DXL	0.034	0.003	0.000	0.716	0.517	0.074
GP36	GP38	QIO	DXL	0.032	0.011	0.000	0.717	0.521	0.072
GP23	GP38	QIO	DXL	0.031	-0.005	0.000	0.711	0.510	0.079
GP13	GP38	QIO	DXL	0.029	0.002	0.000	0.716	0.516	0.072
GP2	GP38	QIO	DXL	0.028	-0.005	0.000	0.709	0.504	0.080
GP24	GP38	QIO	DXL	0.028	0.032	0.000	0.711	0.509	0.074
GP28	GP38	QIO	DXL	0.026	0.004	0.000	0.710	0.503	0.078
GP35	GP38	QIO	DXL	0.026	0.013	0.000	0.718	0.520	0.067
GP22	GP38	QIO	DXL	0.023	0.006	0.000	0.712	0.510	0.075
GP30	GP38	QIO	DXL	0.021	0.015	0.000	0.709	0.507	0.074
GP25	GP38	QIO	DXL	0.021	0.014	0.000	0.710	0.507	0.074
GP33	GP38	QIO	DXL	0.014	0.010	0.000	0.708	0.509	0.069
GP31	GP38	QIO	DXL	-0.003	0.001	0.000	0.705	0.496	0.069
GP38	GP39	DXL	XXL	-0.002	-0.001	0.000	0.705	0.496	0.069
GP37	GP52	LS	LS	0.190	0.097	0.201	0.760	0.582	0.096
GP14	GP52	MIN	LS	-0.018	0.014	0.000	0.681	0.477	0.071
GP14	GP37	MIN	LS	-0.022	0.020	0.000	0.683	0.481	0.068
GP51	GP52	MIN	LS	-0.027	0.031	0.000	0.673	0.470	0.072
GP18	GP52	MIN	LS	-0.028	0.014	0.000	0.676	0.468	0.071
GP18	GP37	MIN	LS	-0.030	0.016	0.000	0.678	0.471	0.070
GP37	GP51	LS	MIN	-0.031	0.024	0.000	0.675	0.472	0.070

GP16	GP37	MIN	LS	-0.042	0.020	0.000	0.676	0.472	0.064
GP16	GP52	MIN	LS	-0.042	0.023	0.000	0.674	0.469	0.064
GP17	GP37	MIN	LS	-0.045	0.027	0.000	0.672	0.467	0.066
GP19	GP52	MIN	LS	-0.046	0.032	0.000	0.668	0.461	0.069
GP17	GP52	MIN	LS	-0.048	0.024	0.000	0.669	0.461	0.068
GP15	GP37	MIN	LS	-0.049	0.029	0.000	0.672	0.468	0.061
GP15	GP52	MIN	LS	-0.049	0.027	0.000	0.670	0.464	0.064
GP19	GP37	MIN	LS	-0.052	0.026	0.000	0.667	0.460	0.067
GP12	GP37	QIN	LS	-0.036	0.033	0.000	0.671	0.477	0.064
GP3	GP52	QIN	LS	-0.037	0.043	0.000	0.663	0.464	0.069
GP3	GP37	QIN	LS	-0.038	0.021	0.000	0.664	0.464	0.070
GP37	GP8	LS	QIN	-0.038	0.033	0.000	0.669	0.474	0.064
GP12	GP52	QIN	LS	-0.040	0.029	0.000	0.667	0.469	0.066
GP52	GP8	LS	QIN	-0.040	0.022	0.000	0.666	0.466	0.067
GP52	GP7	LS	QIN	-0.040	0.036	0.000	0.660	0.461	0.071
GP52	GP6	LS	QIN	-0.042	0.047	0.000	0.657	0.463	0.071
GP37	GP7	LS	QIN	-0.042	0.040	0.000	0.662	0.466	0.068
GP37	GP6	LS	QIN	-0.044	0.040	0.000	0.658	0.466	0.069
GP10	GP52	QIN	LS	-0.045	0.040	0.000	0.661	0.466	0.066
GP37	GP5	LS	QIN	-0.045	0.041	0.000	0.658	0.464	0.070
GP10	GP37	QIN	LS	-0.047	0.040	0.000	0.664	0.471	0.064
GP5	GP52	QIN	LS	-0.047	0.041	0.000	0.655	0.458	0.070
GP4	GP52	QIN	LS	-0.047	0.022	0.000	0.658	0.455	0.071
GP37	GP4	LS	QIN	-0.048	0.036	0.000	0.661	0.462	0.067
GP29	GP52	QIO	LS	0.004	0.012	0.000	0.691	0.488	0.076
GP26	GP52	QIO	LS	0.004	0.020	0.000	0.694	0.494	0.072
GP13	GP52	QIO	LS	0.001	0.010	0.000	0.693	0.491	0.072
GP26	GP37	QIO	LS	-0.001	0.019	0.000	0.694	0.495	0.069
GP36	GP52	QIO	LS	-0.002	0.024	0.000	0.691	0.491	0.071
GP2	GP52	QIO	LS	-0.004	0.015	0.000	0.685	0.478	0.077
GP25	GP52	QIO	LS	-0.006	0.016	0.000	0.687	0.481	0.075
GP29	GP37	QIO	LS	-0.008	0.016	0.000	0.690	0.487	0.072
GP13	GP37	QIO	LS	-0.009	0.009	0.000	0.691	0.489	0.069
GP27	GP52	QIO	LS	-0.009	0.021	0.000	0.682	0.479	0.076
GP24	GP52	QIO	LS	-0.009	0.016	0.000	0.684	0.477	0.076
GP33	GP52	QIO	LS	-0.010	0.022	0.000	0.687	0.488	0.069
GP28	GP52	QIO	LS	-0.010	0.013	0.000	0.683	0.475	0.076
GP36	GP37	QIO	LS	-0.011	0.029	0.000	0.691	0.492	0.067
GP35	GP52	QIO	LS	-0.011	0.014	0.000	0.691	0.491	0.067
GP2	GP37	QIO	LS	-0.012	0.011	0.000	0.685	0.479	0.075
GP35	GP37	QIO	LS	-0.012	0.013	0.000	0.694	0.494	0.065
GP22	GP52	QIO	LS	-0.013	0.014	0.000	0.684	0.479	0.074
GP28	GP37	QIO	LS	-0.013	0.009	0.000	0.684	0.476	0.074
GP27	GP37	QIO	LS	-0.014	0.028	0.000	0.683	0.481	0.073
GP33	GP37	QIO	LS	-0.014	0.022	0.000	0.688	0.492	0.066
GP22	GP37	QIO	LS	-0.014	0.007	0.000	0.687	0.480	0.073

GP24	GP37	QIO	LS	-0.014	0.023	0.000	0.685	0.481	0.072
GP30	GP52	QIO	LS	-0.016	0.019	0.000	0.682	0.478	0.073
GP25	GP37	QIO	LS	-0.017	0.014	0.000	0.684	0.477	0.072
GP23	GP52	QIO	LS	-0.018	0.009	0.000	0.680	0.474	0.075
GP23	GP37	QIO	LS	-0.019	0.014	0.000	0.683	0.477	0.074
GP30	GP37	QIO	LS	-0.024	0.015	0.000	0.681	0.478	0.070
GP31	GP37	QIO	LS	-0.028	0.007	0.000	0.685	0.475	0.069
GP31	GP52	QIO	LS	-0.031	0.007	0.000	0.681	0.469	0.069
GP37	GP39	LS	XXL	-0.029	0.010	0.000	0.686	0.476	0.068
GP39	GP52	XXL	LS	-0.033	0.011	0.000	0.681	0.471	0.068
GP17	GP19	MIN	MIN	0.088	0.032	0.000	0.740	0.553	0.079
GP15	GP17	MIN	MIN	0.079	0.018	0.000	0.741	0.554	0.075
GP15	GP19	MIN	MIN	0.075	0.011	0.000	0.735	0.546	0.077
GP18	GP51	MIN	MIN	0.061	0.020	0.000	0.725	0.531	0.077
GP15	GP16	MIN	MIN	0.061	0.020	0.000	0.735	0.550	0.069
GP16	GP17	MIN	MIN	0.055	0.014	0.000	0.730	0.539	0.073
GP16	GP19	MIN	MIN	0.051	0.009	0.000	0.725	0.530	0.075
GP14	GP18	MIN	MIN	0.050	0.006	0.000	0.723	0.526	0.075
GP16	GP51	MIN	MIN	0.047	0.019	0.000	0.722	0.529	0.074
GP19	GP51	MIN	MIN	0.040	0.014	0.000	0.713	0.516	0.077
GP15	GP51	MIN	MIN	0.037	0.019	0.000	0.717	0.524	0.072
GP17	GP51	MIN	MIN	0.037	0.017	0.000	0.715	0.519	0.075
GP16	GP18	MIN	MIN	0.030	0.014	0.000	0.717	0.519	0.069
GP18	GP19	MIN	MIN	0.028	0.011	0.000	0.711	0.512	0.074
GP14	GP51	MIN	MIN	0.025	0.019	0.000	0.710	0.513	0.073
GP17	GP18	MIN	MIN	0.024	0.005	0.000	0.712	0.511	0.073
GP15	GP18	MIN	MIN	0.019	0.012	0.000	0.712	0.514	0.069
GP14	GP16	MIN	MIN	0.009	0.015	0.000	0.709	0.510	0.065
GP14	GP19	MIN	MIN	0.001	0.007	0.000	0.699	0.495	0.070
GP14	GP15	MIN	MIN	-0.001	0.016	0.000	0.704	0.506	0.064
GP14	GP17	MIN	MIN	-0.005	0.014	0.000	0.700	0.497	0.067
GP12	GP15	QIN	MIN	-0.005	0.018	0.000	0.697	0.506	0.063
GP15	GP4	MIN	QIN	-0.009	0.022	0.000	0.690	0.496	0.066
GP10	GP15	QIN	MIN	-0.010	0.027	0.000	0.692	0.504	0.062
GP15	GP3	MIN	QIN	-0.010	0.022	0.000	0.690	0.497	0.066
GP19	GP3	MIN	QIN	-0.012	0.019	0.000	0.684	0.486	0.071
GP15	GP6	MIN	QIN	-0.012	0.021	0.000	0.683	0.495	0.068
GP15	GP8	MIN	QIN	-0.012	0.023	0.000	0.694	0.501	0.061
GP12	GP17	QIN	MIN	-0.012	0.021	0.000	0.691	0.497	0.063
GP19	GP7	MIN	QIN	-0.012	0.023	0.000	0.683	0.487	0.071
GP12	GP16	QIN	MIN	-0.012	0.023	0.000	0.694	0.501	0.061
GP16	GP8	MIN	QIN	-0.012	0.019	0.000	0.694	0.500	0.061
GP19	GP5	MIN	QIN	-0.012	0.014	0.000	0.680	0.487	0.073
GP15	GP5	MIN	QIN	-0.012	0.021	0.000	0.684	0.493	0.068
GP15	GP7	MIN	QIN	-0.013	0.027	0.000	0.688	0.496	0.066
GP12	GP19	QIN	MIN	-0.014	0.019	0.000	0.689	0.492	0.066

GP19	GP6	MIN	QIN	-0.015	0.021	0.000	0.678	0.486	0.072
GP17	GP7	MIN	QIN	-0.015	0.015	0.000	0.684	0.488	0.071
GP16	GP7	MIN	QIN	-0.015	0.017	0.000	0.686	0.492	0.067
GP19	GP8	MIN	QIN	-0.015	0.018	0.000	0.688	0.494	0.066
GP17	GP3	MIN	QIN	-0.015	0.010	0.000	0.683	0.484	0.071
GP17	GP8	MIN	QIN	-0.016	0.016	0.000	0.689	0.495	0.064
GP19	GP4	MIN	QIN	-0.016	0.017	0.000	0.682	0.483	0.070
GP10	GP17	QIN	MIN	-0.018	0.026	0.000	0.685	0.493	0.064
GP16	GP5	MIN	QIN	-0.018	0.019	0.000	0.682	0.489	0.068
GP10	GP16	QIN	MIN	-0.018	0.023	0.000	0.688	0.497	0.062
GP17	GP6	MIN	QIN	-0.019	0.022	0.000	0.678	0.487	0.070
GP16	GP3	MIN	QIN	-0.019	0.019	0.000	0.686	0.490	0.065
GP10	GP19	QIN	MIN	-0.019	0.022	0.000	0.683	0.489	0.066
GP16	GP6	MIN	QIN	-0.020	0.029	0.000	0.681	0.492	0.066
GP17	GP5	MIN	QIN	-0.020	0.022	0.000	0.679	0.485	0.070
GP17	GP4	MIN	QIN	-0.021	0.020	0.000	0.683	0.485	0.068
GP16	GP4	MIN	QIN	-0.022	0.022	0.000	0.685	0.490	0.064
GP12	GP51	QIN	MIN	-0.024	0.024	0.000	0.682	0.486	0.064
GP18	GP8	MIN	QIN	-0.026	0.010	0.000	0.683	0.483	0.063
GP10	GP51	QIN	MIN	-0.026	0.035	0.000	0.678	0.487	0.065
GP51	GP7	MIN	QIN	-0.027	0.029	0.000	0.675	0.479	0.069
GP4	GP51	QIN	MIN	-0.029	0.020	0.000	0.675	0.477	0.070
GP12	GP18	QIN	MIN	-0.029	0.017	0.000	0.682	0.482	0.062
GP3	GP51	QIN	MIN	-0.030	0.022	0.000	0.675	0.477	0.069
GP5	GP51	QIN	MIN	-0.030	0.038	0.000	0.671	0.478	0.069
GP51	GP6	MIN	QIN	-0.030	0.031	0.000	0.670	0.477	0.070
GP18	GP7	MIN	QIN	-0.032	0.018	0.000	0.674	0.473	0.067
GP51	GP8	MIN	QIN	-0.033	0.020	0.000	0.678	0.481	0.064
GP18	GP4	MIN	QIN	-0.036	0.021	0.000	0.675	0.474	0.065
GP18	GP3	MIN	QIN	-0.036	0.024	0.000	0.675	0.473	0.065
GP12	GP14	QIN	MIN	-0.037	0.018	0.000	0.680	0.481	0.061
GP18	GP5	MIN	QIN	-0.037	0.024	0.000	0.670	0.473	0.068
GP14	GP8	MIN	QIN	-0.037	0.021	0.000	0.680	0.482	0.059
GP10	GP18	QIN	MIN	-0.038	0.017	0.000	0.675	0.476	0.064
GP18	GP6	MIN	QIN	-0.038	0.025	0.000	0.669	0.473	0.067
GP14	GP7	MIN	QIN	-0.042	0.024	0.000	0.670	0.471	0.065
GP10	GP14	QIN	MIN	-0.044	0.028	0.000	0.674	0.479	0.060
GP14	GP4	MIN	QIN	-0.044	0.022	0.000	0.672	0.472	0.063
GP14	GP6	MIN	QIN	-0.045	0.026	0.000	0.666	0.471	0.066
GP14	GP5	MIN	QIN	-0.045	0.026	0.000	0.667	0.470	0.066
GP14	GP3	MIN	QIN	-0.047	0.017	0.000	0.670	0.467	0.065
GP14	GP33	MIN	QIO	0.031	0.022	0.000	0.719	0.527	0.067
GP14	GP28	MIN	QIO	0.008	0.006	0.000	0.704	0.497	0.072
GP18	GP33	MIN	QIO	0.006	0.014	0.000	0.707	0.509	0.065
GP14	GP25	MIN	QIO	0.004	0.000	0.000	0.704	0.499	0.072
GP14	GP24	MIN	QIO	0.001	0.026	0.000	0.701	0.498	0.069

GP14	GP2	MIN	QIO	0.001	0.016	0.000	0.701	0.497	0.071
GP14	GP30	MIN	QIO	0.000	0.008	0.000	0.702	0.498	0.069
GP14	GP35	MIN	QIO	0.000	0.012	0.000	0.710	0.510	0.061
GP18	GP28	MIN	QIO	-0.002	0.003	0.000	0.700	0.491	0.072
GP33	GP51	QIO	MIN	-0.004	0.013	0.000	0.699	0.500	0.066
GP14	GP26	MIN	QIO	-0.005	0.018	0.000	0.703	0.502	0.064
GP14	GP22	MIN	QIO	-0.005	0.009	0.000	0.702	0.497	0.069
GP28	GP51	QIO	MIN	-0.006	0.006	0.000	0.694	0.486	0.073
GP18	GP26	MIN	QIO	-0.006	0.009	0.000	0.701	0.497	0.065
GP13	GP14	QIO	MIN	-0.006	0.007	0.000	0.703	0.499	0.064
GP14	GP29	MIN	QIO	-0.008	0.007	0.000	0.699	0.493	0.068
GP18	GP30	MIN	QIO	-0.009	0.007	0.000	0.698	0.492	0.068
GP14	GP36	MIN	QIO	-0.009	0.015	0.000	0.701	0.502	0.064
GP14	GP27	MIN	QIO	-0.009	0.010	0.000	0.694	0.491	0.070
GP18	GP35	MIN	QIO	-0.010	0.013	0.000	0.704	0.501	0.061
GP13	GP18	QIO	MIN	-0.011	0.006	0.000	0.699	0.493	0.063
GP18	GP25	MIN	QIO	-0.013	0.003	0.000	0.697	0.486	0.068
GP14	GP31	MIN	QIO	-0.014	0.000	0.000	0.701	0.491	0.066
GP18	GP2	MIN	QIO	-0.015	0.006	0.000	0.692	0.482	0.070
GP16	GP33	MIN	QIO	-0.015	0.017	0.000	0.701	0.501	0.059
GP18	GP29	MIN	QIO	-0.016	0.009	0.000	0.695	0.488	0.066
GP18	GP36	MIN	QIO	-0.017	0.010	0.000	0.696	0.491	0.064
GP30	GP51	QIO	MIN	-0.018	0.015	0.000	0.690	0.486	0.068
GP29	GP51	QIO	MIN	-0.018	0.013	0.000	0.691	0.485	0.067
GP22	GP51	QIO	MIN	-0.019	0.010	0.000	0.690	0.483	0.069
GP25	GP51	QIO	MIN	-0.019	0.013	0.000	0.691	0.482	0.069
GP36	GP51	QIO	MIN	-0.019	0.015	0.000	0.692	0.490	0.065
GP15	GP33	MIN	QIO	-0.020	0.015	0.000	0.698	0.499	0.058
GP13	GP51	QIO	MIN	-0.020	0.010	0.000	0.692	0.486	0.064
GP14	GP23	MIN	QIO	-0.021	0.012	0.000	0.691	0.485	0.067
GP16	GP35	MIN	QIO	-0.021	0.015	0.000	0.702	0.500	0.055
GP2	GP51	QIO	MIN	-0.021	0.010	0.000	0.685	0.476	0.072
GP18	GP22	MIN	QIO	-0.021	0.006	0.000	0.694	0.485	0.067
GP18	GP24	MIN	QIO	-0.023	0.016	0.000	0.690	0.483	0.067
GP16	GP28	MIN	QIO	-0.023	0.005	0.000	0.693	0.481	0.065
GP35	GP51	QIO	MIN	-0.023	0.012	0.000	0.695	0.493	0.061
GP26	GP51	QIO	MIN	-0.024	0.016	0.000	0.690	0.488	0.064
GP17	GP33	MIN	QIO	-0.024	0.015	0.000	0.694	0.491	0.061
GP18	GP31	MIN	QIO	-0.024	-0.004	0.000	0.695	0.480	0.065
GP24	GP51	QIO	MIN	-0.025	0.016	0.000	0.685	0.479	0.069
GP19	GP33	MIN	QIO	-0.025	0.015	0.000	0.691	0.488	0.063
GP31	GP51	QIO	MIN	-0.025	-0.006	0.000	0.691	0.479	0.068
GP18	GP27	MIN	QIO	-0.026	0.013	0.000	0.686	0.480	0.068
GP15	GP30	MIN	QIO	-0.027	0.018	0.000	0.691	0.488	0.062
GP16	GP30	MIN	QIO	-0.028	0.014	0.000	0.692	0.487	0.062
GP16	GP25	MIN	QIO	-0.029	0.007	0.000	0.693	0.482	0.063

GP16	GP26	MIN	QIO	-0.030	0.009	0.000	0.693	0.487	0.059
GP16	GP29	MIN	QIO	-0.030	0.008	0.000	0.692	0.483	0.062
GP15	GP25	MIN	QIO	-0.030	-0.002	0.000	0.690	0.478	0.065
GP15	GP26	MIN	QIO	-0.030	0.018	0.000	0.692	0.488	0.057
GP16	GP36	MIN	QIO	-0.031	0.010	0.000	0.693	0.488	0.059
GP15	GP35	MIN	QIO	-0.031	0.014	0.000	0.697	0.494	0.054
GP16	GP24	MIN	QIO	-0.031	0.005	0.000	0.687	0.478	0.064
GP17	GP26	MIN	QIO	-0.031	0.008	0.000	0.690	0.482	0.061
GP19	GP36	MIN	QIO	-0.032	0.009	0.000	0.687	0.481	0.064
GP13	GP16	QIO	MIN	-0.032	0.004	0.000	0.693	0.485	0.058
GP17	GP30	MIN	QIO	-0.032	0.016	0.000	0.688	0.479	0.064
GP19	GP26	MIN	QIO	-0.032	0.016	0.000	0.687	0.482	0.062
GP16	GP31	MIN	QIO	-0.032	-0.002	0.000	0.693	0.481	0.061
GP17	GP28	MIN	QIO	-0.033	0.001	0.000	0.686	0.471	0.068
GP19	GP30	MIN	QIO	-0.033	0.019	0.000	0.684	0.476	0.066
GP15	GP28	MIN	QIO	-0.034	0.004	0.000	0.687	0.473	0.064
GP19	GP28	MIN	QIO	-0.034	0.005	0.000	0.683	0.470	0.069
GP19	GP35	MIN	QIO	-0.034	0.011	0.000	0.691	0.486	0.059
GP17	GP36	MIN	QIO	-0.034	0.014	0.000	0.689	0.483	0.061
GP15	GP36	MIN	QIO	-0.034	0.012	0.000	0.691	0.485	0.058
GP19	GP25	MIN	QIO	-0.035	0.009	0.000	0.685	0.473	0.067
GP17	GP35	MIN	QIO	-0.035	0.008	0.000	0.693	0.485	0.057
GP16	GP2	MIN	QIO	-0.035	0.012	0.000	0.686	0.476	0.063
GP17	GP25	MIN	QIO	-0.035	0.004	0.000	0.686	0.472	0.066
GP15	GP2	MIN	QIO	-0.036	0.013	0.000	0.685	0.478	0.063
GP16	GP22	MIN	QIO	-0.036	0.010	0.000	0.690	0.481	0.061
GP17	GP31	MIN	QIO	-0.037	-0.002	0.000	0.689	0.475	0.064
GP15	GP24	MIN	QIO	-0.037	0.010	0.000	0.685	0.478	0.063
GP13	GP19	QIO	MIN	-0.037	0.007	0.000	0.685	0.476	0.062
GP15	GP31	MIN	QIO	-0.038	0.000	0.000	0.691	0.478	0.060
GP17	GP29	MIN	QIO	-0.038	0.016	0.000	0.685	0.474	0.063
GP13	GP17	QIO	MIN	-0.039	0.014	0.000	0.688	0.480	0.059
GP19	GP22	MIN	QIO	-0.039	-0.003	0.000	0.682	0.469	0.068
GP17	GP24	MIN	QIO	-0.040	0.002	0.000	0.682	0.469	0.066
GP15	GP22	MIN	QIO	-0.040	0.006	0.000	0.687	0.475	0.061
GP17	GP2	MIN	QIO	-0.040	0.021	0.000	0.683	0.474	0.064
GP18	GP23	MIN	QIO	-0.040	0.011	0.000	0.682	0.472	0.065
GP13	GP15	QIO	MIN	-0.040	0.008	0.000	0.688	0.480	0.057
GP19	GP29	MIN	QIO	-0.041	0.010	0.000	0.682	0.471	0.065
GP19	GP2	MIN	QIO	-0.041	0.015	0.000	0.679	0.469	0.068
GP27	GP51	QIO	MIN	-0.041	0.030	0.000	0.677	0.472	0.066
GP17	GP22	MIN	QIO	-0.043	0.010	0.000	0.685	0.472	0.064
GP19	GP31	MIN	QIO	-0.043	0.001	0.000	0.683	0.468	0.064
GP19	GP24	MIN	QIO	-0.043	0.012	0.000	0.678	0.467	0.066
GP15	GP29	MIN	QIO	-0.045	0.008	0.000	0.683	0.474	0.060
GP16	GP27	MIN	QIO	-0.046	0.010	0.000	0.679	0.469	0.063

GP15	GP27	MIN	QIO	-0.047	0.012	0.000	0.678	0.471	0.062
GP15	GP23	MIN	QIO	-0.049	0.020	0.000	0.679	0.473	0.059
GP23	GP51	QIO	MIN	-0.050	0.014	0.000	0.674	0.465	0.066
GP16	GP23	MIN	QIO	-0.051	0.018	0.000	0.679	0.471	0.059
GP19	GP27	MIN	QIO	-0.055	0.024	0.000	0.672	0.464	0.064
GP17	GP27	MIN	QIO	-0.056	0.017	0.000	0.674	0.463	0.063
GP17	GP23	MIN	QIO	-0.060	0.019	0.000	0.674	0.463	0.062
GP19	GP23	MIN	QIO	-0.065	0.022	0.000	0.669	0.458	0.062
GP14	GP39	MIN	XXL	-0.014	0.000	0.000	0.702	0.493	0.065
GP18	GP39	MIN	XXL	-0.023	-0.002	0.000	0.696	0.483	0.064
GP39	GP51	XXL	MIN	-0.025	-0.007	0.000	0.691	0.480	0.067
GP16	GP39	MIN	XXL	-0.031	0.000	0.000	0.694	0.483	0.060
GP15	GP39	MIN	XXL	-0.035	-0.001	0.000	0.693	0.480	0.060
GP17	GP39	MIN	XXL	-0.037	0.002	0.000	0.690	0.478	0.062
GP19	GP39	MIN	XXL	-0.041	-0.005	0.000	0.684	0.469	0.064
GP10	GP5	QIN	QIN	0.305	0.136	0.398	0.818	0.669	0.099
GP6	GP8	QIN	QIN	0.235	0.089	0.285	0.792	0.636	0.091
GP12	GP5	QIN	QIN	0.228	0.070	0.274	0.789	0.631	0.094
GP3	GP6	QIN	QIN	0.204	0.077	0.244	0.772	0.607	0.093
GP5	GP7	QIN	QIN	0.199	0.087	0.228	0.768	0.598	0.096
GP6	GP7	QIN	QIN	0.194	0.082	0.000	0.766	0.600	0.095
GP3	GP5	QIN	QIN	0.191	0.076	0.225	0.766	0.595	0.094
GP10	GP6	QIN	QIN	0.189	0.050	0.000	0.767	0.605	0.091
GP3	GP7	QIN	QIN	0.188	0.075	0.231	0.769	0.601	0.091
GP10	GP7	QIN	QIN	0.186	0.055	0.235	0.770	0.604	0.091
GP4	GP6	QIN	QIN	0.185	0.077	0.000	0.763	0.593	0.093
GP3	GP8	QIN	QIN	0.184	0.053	0.227	0.773	0.605	0.088
GP5	GP6	QIN	QIN	0.184	0.063	0.000	0.757	0.587	0.097
GP7	GP8	QIN	QIN	0.180	0.062	0.228	0.770	0.603	0.088
GP10	GP3	QIN	QIN	0.178	0.052	0.235	0.768	0.603	0.088
GP10	GP12	QIN	QIN	0.178	0.055	0.257	0.777	0.619	0.083
GP4	GP5	QIN	QIN	0.177	0.051	0.000	0.760	0.589	0.094
GP4	GP7	QIN	QIN	0.177	0.053	0.215	0.763	0.591	0.093
GP12	GP7	QIN	QIN	0.176	0.048	0.239	0.771	0.606	0.087
GP10	GP4	QIN	QIN	0.175	0.054	0.235	0.767	0.603	0.088
GP3	GP4	QIN	QIN	0.174	0.042	0.207	0.762	0.588	0.092
GP12	GP6	QIN	QIN	0.174	0.035	0.000	0.764	0.598	0.090
GP4	GP8	QIN	QIN	0.173	0.041	0.225	0.769	0.601	0.087
GP5	GP8	QIN	QIN	0.158	0.043	0.000	0.759	0.589	0.089
GP10	GP8	QIN	QIN	0.157	0.051	0.000	0.767	0.605	0.081
GP12	GP3	QIN	QIN	0.154	0.026	0.215	0.761	0.591	0.086
GP12	GP8	QIN	QIN	0.149	0.043	0.231	0.767	0.602	0.079
GP12	GP4	QIN	QIN	0.143	0.035	0.000	0.757	0.586	0.084
GP33	GP8	QIO	QIN	-0.048	0.026	0.000	0.676	0.481	0.055
GP12	GP33	QIN	QIO	-0.048	0.025	0.000	0.677	0.481	0.055
GP10	GP33	QIN	QIO	-0.052	0.026	0.000	0.671	0.478	0.056

GP3	GP33	QIN	QIO	-0.055	0.022	0.000	0.668	0.468	0.060
GP12	GP35	QIN	QIO	-0.055	0.022	0.000	0.676	0.478	0.051
GP33	GP6	QIO	QIN	-0.056	0.042	0.000	0.665	0.475	0.058
GP33	GP7	QIO	QIN	-0.056	0.021	0.000	0.666	0.467	0.060
GP13	GP8	QIO	QIN	-0.057	0.020	0.000	0.673	0.470	0.054
GP10	GP35	QIN	QIO	-0.057	0.028	0.000	0.672	0.477	0.052
GP12	GP30	QIN	QIO	-0.057	0.027	0.000	0.669	0.467	0.057
GP12	GP26	QIN	QIO	-0.058	0.021	0.000	0.671	0.470	0.055
GP26	GP8	QIO	QIN	-0.059	0.020	0.000	0.670	0.470	0.055
GP35	GP8	QIO	QIN	-0.059	0.020	0.000	0.674	0.475	0.051
GP33	GP5	QIO	QIN	-0.059	0.026	0.000	0.662	0.468	0.061
GP12	GP29	QIN	QIO	-0.060	0.015	0.000	0.667	0.463	0.058
GP12	GP24	QIN	QIO	-0.060	0.017	0.000	0.666	0.462	0.059
GP12	GP13	QIN	QIO	-0.061	0.016	0.000	0.671	0.468	0.054
GP12	GP36	QIN	QIO	-0.061	0.022	0.000	0.669	0.468	0.054
GP33	GP4	QIO	QIN	-0.061	0.029	0.000	0.666	0.467	0.058
GP26	GP7	QIO	QIN	-0.061	0.023	0.000	0.663	0.463	0.060
GP36	GP8	QIO	QIN	-0.061	0.024	0.000	0.669	0.469	0.054
GP24	GP3	QIO	QIN	-0.062	0.021	0.000	0.661	0.455	0.064
GP26	GP3	QIO	QIN	-0.062	0.018	0.000	0.664	0.461	0.060
GP12	GP28	QIN	QIO	-0.062	0.012	0.000	0.665	0.455	0.061
GP10	GP26	QIN	QIO	-0.062	0.026	0.000	0.665	0.467	0.055
GP3	GP35	QIN	QIO	-0.063	0.016	0.000	0.668	0.466	0.057
GP13	GP3	QIO	QIN	-0.064	0.018	0.000	0.665	0.458	0.058
GP10	GP30	QIN	QIO	-0.064	0.028	0.000	0.662	0.463	0.059
GP30	GP7	QIO	QIN	-0.064	0.025	0.000	0.660	0.458	0.062
GP26	GP6	QIO	QIN	-0.065	0.030	0.000	0.660	0.464	0.060
GP35	GP5	QIO	QIN	-0.065	0.024	0.000	0.663	0.466	0.056
GP28	GP8	QIO	QIN	-0.066	0.009	0.000	0.663	0.452	0.060
GP10	GP24	QIN	QIO	-0.066	0.022	0.000	0.659	0.457	0.060
GP2	GP8	QIO	QIN	-0.066	0.014	0.000	0.662	0.456	0.061
GP30	GP8	QIO	QIN	-0.066	0.020	0.000	0.665	0.462	0.058
GP35	GP7	QIO	QIN	-0.067	0.023	0.000	0.665	0.466	0.055
GP24	GP8	QIO	QIN	-0.067	0.019	0.000	0.663	0.457	0.059
GP29	GP3	QIO	QIN	-0.067	0.014	0.000	0.660	0.453	0.062
GP35	GP4	QIO	QIN	-0.067	0.016	0.000	0.666	0.463	0.055
GP28	GP3	QIO	QIN	-0.067	0.017	0.000	0.659	0.447	0.064
GP13	GP7	QIO	QIN	-0.067	0.020	0.000	0.663	0.458	0.059
GP3	GP30	QIN	QIO	-0.067	0.024	0.000	0.660	0.457	0.062
GP10	GP13	QIN	QIO	-0.067	0.022	0.000	0.665	0.464	0.055
GP13	GP6	QIO	QIN	-0.067	0.026	0.000	0.660	0.461	0.059
GP25	GP8	QIO	QIN	-0.068	0.009	0.000	0.664	0.454	0.059
GP2	GP3	QIO	QIN	-0.068	0.021	0.000	0.657	0.449	0.065
GP25	GP3	QIO	QIN	-0.068	0.010	0.000	0.659	0.449	0.064
GP26	GP5	QIO	QIN	-0.068	0.016	0.000	0.656	0.456	0.062
GP12	GP2	QIN	QIO	-0.068	0.014	0.000	0.661	0.455	0.060

GP2	GP7	QIO	QIN	-0.068	0.018	0.000	0.656	0.450	0.066
GP10	GP29	QIN	QIO	-0.068	0.029	0.000	0.661	0.461	0.057
GP10	GP2	QIN	QIO	-0.068	0.027	0.000	0.658	0.455	0.061
GP26	GP4	QIO	QIN	-0.068	0.029	0.000	0.662	0.460	0.056
GP24	GP6	QIO	QIN	-0.069	0.028	0.000	0.653	0.452	0.064
GP24	GP7	QIO	QIN	-0.069	0.023	0.000	0.656	0.450	0.064
GP29	GP7	QIO	QIN	-0.069	0.026	0.000	0.659	0.456	0.061
GP12	GP25	QIN	QIO	-0.069	0.011	0.000	0.664	0.455	0.059
GP3	GP36	QIN	QIO	-0.069	0.019	0.000	0.661	0.457	0.058
GP29	GP8	QIO	QIN	-0.069	0.015	0.000	0.664	0.458	0.057
GP35	GP6	QIO	QIN	-0.069	0.026	0.000	0.661	0.466	0.056
GP31	GP8	QIO	QIN	-0.069	0.007	0.000	0.667	0.456	0.056
GP12	GP22	QIN	QIO	-0.069	0.008	0.000	0.663	0.454	0.058
GP12	GP31	QIN	QIO	-0.069	0.009	0.000	0.667	0.456	0.055
GP10	GP28	QIN	QIO	-0.069	0.016	0.000	0.659	0.451	0.061
GP24	GP5	QIO	QIN	-0.070	0.022	0.000	0.652	0.448	0.065
GP31	GP7	QIO	QIN	-0.070	0.007	0.000	0.661	0.451	0.062
GP30	GP6	QIO	QIN	-0.070	0.031	0.000	0.655	0.457	0.062
GP13	GP4	QIO	QIN	-0.070	0.020	0.000	0.663	0.457	0.057
GP28	GP4	QIO	QIN	-0.070	0.016	0.000	0.658	0.446	0.063
GP10	GP36	QIN	QIO	-0.070	0.029	0.000	0.662	0.465	0.054
GP28	GP6	QIO	QIN	-0.070	0.015	0.000	0.652	0.444	0.066
GP30	GP4	QIO	QIN	-0.071	0.019	0.000	0.658	0.452	0.062
GP2	GP5	QIO	QIN	-0.071	0.021	0.000	0.652	0.448	0.066
GP22	GP8	QIO	QIN	-0.071	0.017	0.000	0.663	0.456	0.057
GP36	GP7	QIO	QIN	-0.071	0.027	0.000	0.659	0.457	0.058
GP36	GP4	QIO	QIN	-0.071	0.029	0.000	0.661	0.458	0.056
GP28	GP5	QIO	QIN	-0.072	0.019	0.000	0.652	0.444	0.065
GP2	GP4	QIO	QIN	-0.072	0.016	0.000	0.656	0.446	0.064
GP29	GP5	QIO	QIN	-0.072	0.018	0.000	0.653	0.450	0.064
GP13	GP5	QIO	QIN	-0.072	0.020	0.000	0.657	0.455	0.059
GP2	GP6	QIO	QIN	-0.073	0.023	0.000	0.651	0.447	0.066
GP30	GP5	QIO	QIN	-0.073	0.026	0.000	0.652	0.451	0.064
GP25	GP7	QIO	QIN	-0.073	0.018	0.000	0.656	0.447	0.062
GP3	GP31	QIN	QIO	-0.073	0.006	0.000	0.661	0.448	0.060
GP36	GP5	QIO	QIN	-0.073	0.027	0.000	0.656	0.458	0.059
GP28	GP7	QIO	QIN	-0.074	0.012	0.000	0.654	0.442	0.065
GP24	GP4	QIO	QIN	-0.074	0.022	0.000	0.655	0.447	0.062
GP36	GP6	QIO	QIN	-0.075	0.030	0.000	0.655	0.457	0.059
GP10	GP25	QIN	QIO	-0.075	0.020	0.000	0.657	0.452	0.058
GP22	GP3	QIO	QIN	-0.075	0.019	0.000	0.657	0.447	0.061
GP31	GP6	QIO	QIN	-0.075	0.008	0.000	0.656	0.448	0.062
GP29	GP6	QIO	QIN	-0.075	0.031	0.000	0.653	0.454	0.060
GP25	GP4	QIO	QIN	-0.076	0.010	0.000	0.656	0.444	0.062
GP29	GP4	QIO	QIN	-0.076	0.018	0.000	0.657	0.448	0.060
GP25	GP6	QIO	QIN	-0.076	0.019	0.000	0.651	0.446	0.064

GP22	GP4	QIO	QIN	-0.077	0.016	0.000	0.658	0.447	0.061
GP10	GP31	QIN	QIO	-0.077	0.008	0.000	0.660	0.451	0.057
GP25	GP5	QIO	QIN	-0.077	0.020	0.000	0.651	0.446	0.064
GP22	GP7	QIO	QIN	-0.077	0.016	0.000	0.656	0.448	0.062
GP27	GP8	QIO	QIN	-0.078	0.024	0.000	0.656	0.452	0.058
GP23	GP3	QIO	QIN	-0.078	0.022	0.000	0.653	0.446	0.062
GP12	GP23	QIN	QIO	-0.079	0.027	0.000	0.657	0.454	0.056
GP27	GP3	QIO	QIN	-0.079	0.026	0.000	0.651	0.446	0.063
GP10	GP23	QIN	QIO	-0.079	0.029	0.000	0.654	0.454	0.058
GP12	GP27	QIN	QIO	-0.079	0.026	0.000	0.655	0.453	0.056
GP31	GP4	QIO	QIN	-0.080	0.009	0.000	0.659	0.446	0.059
GP10	GP22	QIN	QIO	-0.080	0.019	0.000	0.656	0.450	0.057
GP31	GP5	QIO	QIN	-0.080	0.015	0.000	0.654	0.447	0.060
GP27	GP7	QIO	QIN	-0.080	0.032	0.000	0.649	0.447	0.062
GP10	GP27	QIN	QIO	-0.081	0.029	0.000	0.651	0.452	0.059
GP22	GP6	QIO	QIN	-0.081	0.016	0.000	0.650	0.444	0.063
GP23	GP8	QIO	QIN	-0.081	0.020	0.000	0.656	0.450	0.057
GP27	GP5	QIO	QIN	-0.082	0.044	0.000	0.647	0.449	0.061
GP23	GP7	QIO	QIN	-0.082	0.030	0.000	0.650	0.446	0.061
GP22	GP5	QIO	QIN	-0.082	0.024	0.000	0.651	0.446	0.062
GP27	GP6	QIO	QIN	-0.084	0.031	0.000	0.645	0.447	0.064
GP23	GP6	QIO	QIN	-0.084	0.036	0.000	0.647	0.450	0.061
GP27	GP4	QIO	QIN	-0.087	0.030	0.000	0.649	0.444	0.060
GP23	GP5	QIO	QIN	-0.089	0.041	0.000	0.645	0.444	0.060
GP23	GP4	QIO	QIN	-0.092	0.028	0.000	0.648	0.441	0.059
GP12	GP39	QIN	XXL	-0.068	0.006	0.000	0.668	0.458	0.055
GP39	GP8	XXL	QIN	-0.069	0.006	0.000	0.668	0.458	0.055
GP39	GP7	XXL	QIN	-0.070	0.010	0.000	0.661	0.453	0.061
GP3	GP39	QIN	XXL	-0.074	0.006	0.000	0.660	0.449	0.060
GP39	GP6	XXL	QIN	-0.074	0.009	0.000	0.656	0.451	0.062
GP10	GP39	QIN	XXL	-0.075	0.010	0.000	0.661	0.454	0.056
GP39	GP4	XXL	QIN	-0.079	0.011	0.000	0.659	0.448	0.058
GP39	GP5	XXL	QIN	-0.081	0.014	0.000	0.654	0.447	0.060
GP23	GP27	QIO	QIO	0.274	0.196	0.422	0.824	0.679	0.085
GP22	GP31	QIO	QIO	0.100	-0.020	0.173	0.754	0.554	0.084
GP25	GP28	QIO	QIO	0.078	0.009	0.117	0.738	0.540	0.082
GP24	GP30	QIO	QIO	0.076	0.014	0.000	0.734	0.541	0.080
GP23	GP31	QIO	QIO	0.066	-0.007	0.111	0.735	0.536	0.079
GP13	GP2	QIO	QIO	0.065	0.010	0.000	0.734	0.538	0.076
GP22	GP24	QIO	QIO	0.059	0.002	0.000	0.729	0.529	0.079
GP22	GP30	QIO	QIO	0.058	0.000	0.000	0.729	0.530	0.078
GP29	GP30	QIO	QIO	0.057	0.001	0.000	0.728	0.530	0.078
GP2	GP36	QIO	QIO	0.055	0.017	0.000	0.728	0.533	0.075
GP2	GP22	QIO	QIO	0.051	0.012	0.000	0.724	0.522	0.078
GP27	GP31	QIO	QIO	0.050	-0.008	0.000	0.726	0.526	0.078
GP2	GP29	QIO	QIO	0.049	0.011	0.000	0.723	0.524	0.078

GP24	GP29	QIO	QIO	0.047	0.009	0.000	0.723	0.525	0.076
GP30	GP36	QIO	QIO	0.046	-0.001	0.000	0.725	0.528	0.074
GP2	GP30	QIO	QIO	0.045	0.010	0.000	0.720	0.520	0.078
GP28	GP36	QIO	QIO	0.044	-0.004	0.000	0.722	0.522	0.076
GP24	GP35	QIO	QIO	0.044	0.009	0.000	0.728	0.532	0.069
GP29	GP35	QIO	QIO	0.043	0.013	0.000	0.730	0.537	0.067
GP29	GP36	QIO	QIO	0.043	0.011	0.000	0.726	0.530	0.072
GP24	GP28	QIO	QIO	0.040	-0.006	0.000	0.716	0.510	0.080
GP26	GP28	QIO	QIO	0.039	0.003	0.000	0.720	0.518	0.074
GP26	GP30	QIO	QIO	0.038	0.016	0.000	0.722	0.528	0.070
GP2	GP24	QIO	QIO	0.038	0.006	0.000	0.715	0.512	0.079
GP22	GP36	QIO	QIO	0.038	0.017	0.000	0.725	0.528	0.070
GP13	GP35	QIO	QIO	0.037	0.018	0.000	0.732	0.539	0.062
GP13	GP24	QIO	QIO	0.037	0.001	0.000	0.720	0.519	0.072
GP13	GP29	QIO	QIO	0.037	0.012	0.000	0.725	0.526	0.069
GP13	GP30	QIO	QIO	0.037	0.003	0.000	0.722	0.523	0.071
GP30	GP35	QIO	QIO	0.035	0.015	0.000	0.726	0.531	0.067
GP13	GP26	QIO	QIO	0.035	0.011	0.000	0.725	0.530	0.066
GP22	GP25	QIO	QIO	0.034	0.005	0.000	0.721	0.517	0.076
GP24	GP25	QIO	QIO	0.034	0.008	0.000	0.716	0.513	0.076
GP28	GP33	QIO	QIO	0.033	0.008	0.000	0.718	0.520	0.072
GP24	GP36	QIO	QIO	0.033	0.003	0.000	0.718	0.519	0.073
GP2	GP26	QIO	QIO	0.033	0.018	0.000	0.717	0.518	0.072
GP26	GP29	QIO	QIO	0.033	0.006	0.000	0.720	0.521	0.071
GP13	GP36	QIO	QIO	0.033	0.011	0.000	0.724	0.529	0.067
GP2	GP25	QIO	QIO	0.033	0.003	0.000	0.716	0.512	0.078
GP2	GP35	QIO	QIO	0.032	0.007	0.000	0.721	0.521	0.070
GP35	GP36	QIO	QIO	0.031	0.010	0.000	0.727	0.533	0.063
GP30	GP33	QIO	QIO	0.031	0.015	0.000	0.717	0.523	0.068
GP25	GP35	QIO	QIO	0.031	0.010	0.000	0.725	0.527	0.067
GP28	GP30	QIO	QIO	0.031	0.005	0.000	0.715	0.512	0.075
GP25	GP33	QIO	QIO	0.029	0.009	0.000	0.718	0.518	0.069
GP26	GP36	QIO	QIO	0.029	0.012	0.000	0.722	0.526	0.067
GP22	GP28	QIO	QIO	0.029	0.003	0.000	0.716	0.508	0.076
GP22	GP29	QIO	QIO	0.028	0.011	0.000	0.718	0.516	0.072
GP13	GP25	QIO	QIO	0.028	-0.001	0.000	0.720	0.516	0.071
GP2	GP28	QIO	QIO	0.028	0.007	0.000	0.712	0.505	0.078
GP25	GP29	QIO	QIO	0.027	0.007	0.000	0.716	0.512	0.074
GP28	GP29	QIO	QIO	0.027	0.004	0.000	0.713	0.508	0.074
GP25	GP36	QIO	QIO	0.026	0.008	0.000	0.717	0.516	0.071
GP26	GP35	QIO	QIO	0.025	0.009	0.000	0.724	0.529	0.063
GP24	GP26	QIO	QIO	0.024	0.011	0.000	0.714	0.512	0.071
GP13	GP27	QIO	QIO	0.024	0.007	0.000	0.714	0.516	0.071
GP24	GP27	QIO	QIO	0.023	0.015	0.000	0.707	0.506	0.076
GP26	GP27	QIO	QIO	0.022	0.014	0.000	0.711	0.512	0.072
GP13	GP22	QIO	QIO	0.022	-0.003	0.000	0.718	0.512	0.070

GP22	GP35	QIO	QIO	0.022	0.008	0.000	0.722	0.520	0.066
GP25	GP30	QIO	QIO	0.021	0.006	0.000	0.712	0.508	0.074
GP25	GP26	QIO	QIO	0.021	0.005	0.000	0.715	0.513	0.070
GP24	GP33	QIO	QIO	0.020	0.015	0.000	0.712	0.513	0.069
GP33	GP35	QIO	QIO	0.018	0.014	0.000	0.721	0.528	0.060
GP28	GP35	QIO	QIO	0.018	0.000	0.000	0.716	0.513	0.068
GP29	GP33	QIO	QIO	0.016	0.019	0.000	0.714	0.519	0.065
GP23	GP24	QIO	QIO	0.016	0.014	0.000	0.707	0.505	0.074
GP13	GP33	QIO	QIO	0.016	0.011	0.000	0.715	0.519	0.063
GP27	GP29	QIO	QIO	0.015	0.022	0.000	0.707	0.508	0.072
GP22	GP33	QIO	QIO	0.015	0.009	0.000	0.713	0.514	0.068
GP22	GP27	QIO	QIO	0.015	-0.011	0.000	0.707	0.501	0.077
GP27	GP36	QIO	QIO	0.015	0.008	0.000	0.707	0.508	0.072
GP13	GP23	QIO	QIO	0.015	0.008	0.000	0.712	0.510	0.069
GP13	GP28	QIO	QIO	0.015	0.002	0.000	0.712	0.507	0.069
GP2	GP31	QIO	QIO	0.014	-0.002	0.000	0.713	0.503	0.072
GP2	GP23	QIO	QIO	0.014	0.006	0.000	0.705	0.499	0.076
GP22	GP26	QIO	QIO	0.013	0.007	0.000	0.713	0.511	0.068
GP26	GP33	QIO	QIO	0.013	0.021	0.000	0.714	0.519	0.061
GP13	GP31	QIO	QIO	0.013	0.000	0.000	0.718	0.512	0.066
GP33	GP36	QIO	QIO	0.012	0.019	0.000	0.713	0.518	0.062
GP2	GP27	QIO	QIO	0.012	0.014	0.000	0.703	0.499	0.076
GP31	GP36	QIO	QIO	0.012	0.012	0.000	0.716	0.511	0.065
GP2	GP33	QIO	QIO	0.010	0.009	0.000	0.707	0.507	0.069
GP23	GP26	QIO	QIO	0.010	0.020	0.000	0.709	0.510	0.068
GP25	GP27	QIO	QIO	0.010	0.010	0.000	0.704	0.499	0.074
GP29	GP31	QIO	QIO	0.009	0.000	0.000	0.713	0.504	0.069
GP27	GP35	QIO	QIO	0.007	0.008	0.000	0.710	0.512	0.066
GP24	GP31	QIO	QIO	0.007	0.003	0.000	0.710	0.499	0.070
GP31	GP35	QIO	QIO	0.007	0.002	0.000	0.718	0.512	0.062
GP23	GP35	QIO	QIO	0.006	0.014	0.000	0.711	0.513	0.064
GP23	GP36	QIO	QIO	0.006	0.011	0.000	0.706	0.504	0.069
GP30	GP31	QIO	QIO	0.005	-0.006	0.000	0.709	0.500	0.071
GP27	GP28	QIO	QIO	0.004	0.012	0.000	0.699	0.493	0.073
GP23	GP25	QIO	QIO	0.003	-0.004	0.000	0.702	0.494	0.075
GP23	GP29	QIO	QIO	0.003	0.021	0.000	0.704	0.502	0.069
GP23	GP28	QIO	QIO	0.002	-0.006	0.000	0.700	0.490	0.076
GP22	GP23	QIO	QIO	0.002	0.010	0.000	0.705	0.498	0.072
GP26	GP31	QIO	QIO	0.001	0.005	0.000	0.711	0.504	0.065
GP25	GP31	QIO	QIO	-0.002	-0.005	0.000	0.707	0.493	0.069
GP28	GP31	QIO	QIO	-0.004	-0.002	0.000	0.705	0.492	0.069
GP27	GP30	QIO	QIO	-0.004	0.022	0.000	0.698	0.497	0.070
GP23	GP30	QIO	QIO	-0.005	0.018	0.000	0.699	0.496	0.069
GP31	GP33	QIO	QIO	-0.005	0.004	0.000	0.707	0.502	0.063
GP27	GP33	QIO	QIO	-0.008	0.008	0.000	0.696	0.497	0.068
GP23	GP33	QIO	QIO	-0.011	0.012	0.000	0.698	0.497	0.065

GP31	GP39	QIO	XXL	0.388	0.430	0.775	0.925	0.861	0.047
GP22	GP39	QIO	XXL	0.098	-0.017	0.171	0.754	0.555	0.083
GP23	GP39	QIO	XXL	0.064	-0.008	0.112	0.734	0.536	0.078
GP27	GP39	QIO	XXL	0.049	-0.012	0.000	0.726	0.526	0.078
GP2	GP39	QIO	XXL	0.014	-0.004	0.000	0.713	0.504	0.071
GP36	GP39	QIO	XXL	0.012	0.011	0.000	0.717	0.513	0.065
GP13	GP39	QIO	XXL	0.012	0.000	0.000	0.718	0.513	0.064
GP29	GP39	QIO	XXL	0.008	-0.002	0.000	0.713	0.505	0.068
GP35	GP39	QIO	XXL	0.007	0.001	0.000	0.719	0.514	0.061
GP24	GP39	QIO	XXL	0.006	-0.001	0.000	0.710	0.500	0.069
GP30	GP39	QIO	XXL	0.004	-0.006	0.000	0.709	0.501	0.069
GP26	GP39	QIO	XXL	0.001	0.005	0.000	0.712	0.506	0.064
GP33	GP39	QIO	XXL	-0.003	0.007	0.000	0.710	0.506	0.062
GP28	GP39	QIO	XXL	-0.003	-0.003	0.000	0.706	0.494	0.068

f_{jk}-IV = genomic coancestry coefficient, d_{jk}-IV = dominance relationship, IBD = probability of alleles identical by descent, IBS = probability of alleles identical by state, IBG = probability of SNP loci identical by genotype , NSG = probability of non-shared genotypes that have no common alleles between two genotypes. f_{jk}-IV and d_{jk}-IV were calculated based on the diagonal elements of Definition IV of genomic additive relationship matrix. DXL = Daxiangling, LS = Liangshan, MIN = Minshan, QIN = Qinling, QIO=Qionglai, XXL = Xiaoxiangling.
