# Supplementary material for: Genomic Inbreeding and Relatedness in Wild Panda Populations
Source: PLoS One. 2016 Aug 5;11(8):e0160496. doi: 10.1371/journal.pone.0160496 (PMC4975500; doi:10.1371/journal.pone.0160496)
Supplement: S3 Table — (PDF) [file pone.0160496.s006.pdf]

**S3 Table. Mean, standard deviation and range of pairwise genomic relatedness and similarity measures within and between habitats calculated from S2 Table.**

| Variable | N | Mean | Standard deviation | Minimum | Maximum |
|---|---|---|---|---|---|
| | | | **Liangshan** | | |
| $f_m$ | 1 | 0.148 | . | 0.148 | 0.148 |
| $d_m$ | 1 | 0.073 | . | 0.073 | 0.073 |
| IBD | 1 | 0.201 | . | 0.201 | 0.201 |
| IBS | 1 | 0.76 | . | 0.76 | 0.76 |
| IBG | 1 | 0.582 | . | 0.582 | 0.582 |
| NSG | 1 | 0.096 | . | 0.096 | 0.096 |
| | | | **Minshan** | | |
| $f_m$ | 21 | 0.034 | 0.024 | -0.005 | 0.078 |
| $d_m$ | 21 | 0.024 | 0.008 | 0.011 | 0.042 |
| IBD | 21 | 0.000 | 0.000 | 0.000 | 0.000 |
| IBS | 21 | 0.719 | 0.012 | 0.699 | 0.741 |
| IBG | 21 | 0.524 | 0.017 | 0.495 | 0.554 |
| NSG | 21 | 0.072 | 0.004 | 0.064 | 0.079 |
| | | | **Qinling** | | |
| $f_m$ | 28 | 0.149 | 0.024 | 0.119 | 0.244 |
| $d_m$ | 28 | 0.063 | 0.014 | 0.046 | 0.113 |
| IBD | 28 | 0.166 | 0.121 | 0.000 | 0.398 |
| IBS | 28 | 0.770 | 0.012 | 0.757 | 0.818 |
| IBG | 28 | 0.604 | 0.017 | 0.586 | 0.669 |
| NSG | 28 | 0.090 | 0.005 | 0.079 | 0.099 |
| | | | **Qionglai** | | |
| $f_m$ | 105 | 0.025 | 0.028 | -0.010 | 0.237 |
| $d_m$ | 105 | 0.019 | 0.017 | 0.000 | 0.181 |
| IBD | 105 | 0.008 | 0.047 | 0.000 | 0.422 |
| IBS | 105 | 0.718 | 0.014 | 0.696 | 0.824 |
| IBG | 105 | 0.517 | 0.020 | 0.490 | 0.679 |
| NSG | 105 | 0.071 | 0.005 | 0.060 | 0.085 |
| | | | **Daxiangling-Liangshan** | | |
| $f_m$ | 2 | 0.005 | 0.010 | -0.002 | 0.012 |
| $d_m$ | 2 | 0.025 | 0.001 | 0.024 | 0.026 |
| IBD | 2 | 0.000 | 0.000 | 0.000 | 0.000 |
| IBS | 2 | 0.691 | 0.002 | 0.689 | 0.692 |
| IBG | 2 | 0.489 | 0.001 | 0.488 | 0.490 |
| NSG | 2 | 0.076 | 0.004 | 0.073 | 0.078 |
| | | | **Daxiangling-Minshan** | | |
| $f_m$ | 3 | -0.022 | 0.013 | -0.034 | 0.003 |

| | | | | | |
|---|---|---|---|---|---|
| $d_m$ | 3 | 0.013 | 0.004 | 0.008 | 0.017 |
| IBD | 3 | 0.000 | 0.000 | 0.000 | 0.000 |
| IBS | 3 | 0.682 | 0.012 | 0.671 | 0.695 |
| IBG | 3 | 0.473 | 0.008 | 0.466 | 0.482 |
| NSG | 3 | 0.066 | 0.002 | 0.065 | 0.068 |
| | | | **Daxiangling-Qinling** | | |
| $f_m$ | 8 | -0.058 | 0.003 | -0.062 | -0.054 |
| $d_m$ | 8 | 0.014 | 0.004 | 0.011 | 0.021 |
| IBD | 8 | 0.000 | 0.000 | 0.000 | 0.000 |
| IBS | 8 | 0.657 | 0.005 | 0.650 | 0.665 |
| IBG | 8 | 0.454 | 0.004 | 0.450 | 0.461 |
| NSG | 8 | -0.069 | 0.004 | -0.076 | -0.064 |
| | | | **Daxiangling-Qionglai** | | |
| $f_m$ | 15 | 0.022 | 0.009 | -0.003 | 0.034 |
| $d_m$ | 15 | 0.017 | 0.007 | 0.006 | 0.033 |
| IBD | 15 | 0.000 | 0.000 | 0.000 | 0.000 |
| IBS | 15 | 0.712 | 0.004 | 0.705 | 0.718 |
| IBG | 15 | 0.511 | 0.008 | 0.496 | 0.522 |
| NSG | 15 | 0.074 | 0.004 | 0.067 | 0.080 |
| | | | **Daxiangling-Xiaoxiangling** | | |
| $f_m$ | 1 | -0.003 | . | -0.003 | -0.003 |
| $d_m$ | 1 | 0.007 | . | 0.007 | 0.007 |
| IBD | 1 | 0.000 | . | 0.000 | 0.000 |
| IBS | 1 | 0.705 | . | 0.705 | 0.705 |
| IBG | 1 | 0.496 | . | 0.496 | 0.496 |
| NSG | 1 | 0.069 | . | 0.069 | 0.069 |
| | | | **Liangshan-Minshan** | | |
| $f_m$ | 14 | -0.032 | 0.010 | -0.044 | -0.016 |
| $d_m$ | 14 | 0.018 | 0.004 | 0.012 | 0.024 |
| IBD | 14 | 0.000 | 0.000 | 0.000 | 0.000 |
| IBS | 14 | 0.674 | 0.005 | 0.667 | 0.683 |
| IBG | 14 | 0.469 | 0.006 | 0.460 | 0.481 |
| NSG | 14 | 0.068 | 0.003 | 0.061 | 0.072 |
| | | | **Liangshan-Qinling** | | |
| $f_m$ | 16 | -0.034 | 0.003 | -0.038 | -0.029 |
| $d_m$ | 16 | 0.023 | 0.005 | 0.014 | 0.028 |
| IBD | 16 | 0.000 | 0.000 | 0.000 | 0.000 |
| IBS | 16 | 0.662 | 0.005 | 0.655 | 0.671 |
| IBG | 16 | 0.465 | 0.006 | 0.455 | 0.477 |
| NSG | 16 | 0.068 | 0.003 | 0.064 | 0.071 |
| | | | **Liangshan-Qionglai** | | |
| $f_m$ | 30 | -0.010 | 0.007 | -0.027 | 0.004 |
| $d_m$ | 30 | 0.016 | 0.005 | 0.010 | 0.028 |

| | | | | | |
|---|---|---|---|---|---|
| IBD | 30 | 0.000 | 0.000 | 0.000 | 0.000 |
| IBS | 30 | 0.687 | 0.004 | 0.680 | 0.694 |
| IBG | 30 | 0.483 | 0.007 | 0.469 | 0.495 |
| NSG | 30 | 0.072 | 0.003 | 0.065 | 0.077 |
| **Liangshan-Xiaoxiangling** | | | | | |
| $f_m$ | 1 | -0.028 | 0.002 | -0.029 | -0.026 |
| $d_m$ | 1 | 0.013 | 0.001 | 0.012 | 0.014 |
| IBD | 1 | 0.000 | . | 0.000 | 0.000 |
| IBS | 1 | 0.705 | . | 0.705 | 0.705 |
| IBG | 1 | 0.496 | . | 0.496 | 0.496 |
| NSG | 1 | 0.069 | . | 0.069 | 0.069 |
| **Minshan-Qinling** | | | | | |
| $f_m$ | 56 | -0.020 | 0.010 | -0.040 | -0.004 |
| $d_m$ | 56 | 0.020 | 0.005 | 0.009 | 0.032 |
| IBD | 56 | 0.000 | 0.000 | 0.000 | 0.000 |
| IBS | 56 | 0.681 | 0.008 | 0.666 | 0.697 |
| IBG | 56 | 0.486 | 0.010 | 0.467 | 0.506 |
| NSG | 56 | 0.066 | 0.003 | 0.059 | 0.073 |
| **Minshan-Qionglai** | | | | | |
| $f_m$ | 105 | -0.024 | 0.014 | -0.057 | 0.028 |
| $d_m$ | 105 | 0.012 | 0.006 | -0.001 | 0.030 |
| IBD | 105 | 0.000 | 0.000 | 0.000 | 0.000 |
| IBS | 105 | 0.691 | 0.009 | 0.669 | 0.719 |
| IBG | 105 | 0.483 | 0.012 | 0.458 | 0.527 |
| NSG | 105 | 0.064 | 0.004 | 0.054 | 0.073 |
| **Minshan-Xiaoxiangling** | | | | | |
| $f_m$ | 7 | -0.028 | 0.009 | -0.039 | -0.014 |
| $d_m$ | 7 | 0.001 | 0.004 | -0.005 | 0.006 |
| IBD | 7 | 0.000 | 0.000 | 0.000 | 0.000 |
| IBS | 7 | 0.693 | 0.006 | 0.684 | 0.702 |
| IBG | 7 | 0.481 | 0.007 | 0.469 | 0.493 |
| NSG | 7 | 0.063 | 0.003 | 0.060 | 0.067 |
| **Qinling-Qionglai** | | | | | |
| $f_m$ | 120 | -0.059 | 0.006 | -0.077 | -0.042 |
| $d_m$ | 120 | -0.059 | 0.006 | -0.077 | -0.042 |
| IBD | 120 | 0.000 | 0.000 | 0.000 | 0.000 |
| IBS | 120 | 0.660 | 0.007 | 0.645 | 0.677 |
| IBG | 120 | 0.456 | 0.009 | 0.441 | 0.481 |
| NSG | 120 | 0.060 | 0.003 | 0.051 | 0.066 |
| **Qinling-Xiaoxiangling** | | | | | |
| $f_m$ | 8 | -0.066 | 0.003 | -0.070 | -0.062 |
| $d_m$ | 8 | 0.002 | 0.002 | 0.000 | 0.006 |
| IBD | 8 | 0.000 | 0.000 | 0.000 | 0.000 |

| | | | | | |
|---|---|---|---|---|---|
| IBS | 8 | 0.661 | 0.005 | 0.654 | 0.668 |
| IBG | 8 | 0.452 | 0.004 | 0.447 | 0.458 |
| NSG | 8 | 0.058 | 0.003 | 0.055 | 0.062 |
| | | **Qionglai-Xiaoxiangling** | | | |
| $f_m$ | 15 | 0.042 | 0.100 | -0.005 | 0.388 |
| $d_m$ | 15 | 0.044 | 0.132 | 0.001 | 0.523 |
| IBD | 15 | 0.071 | 0.201 | 0.000 | 0.775 |
| IBS | 15 | 0.732 | 0.055 | 0.706 | 0.925 |
| IBG | 15 | 0.535 | 0.092 | 0.494 | 0.861 |
| NSG | 15 | 0.068 | 0.008 | 0.047 | 0.083 |

$f_m$ = average genomic coancestry coefficient, $d_m$ = average dominance relationship, IBD = probability of alleles identical by descent, IBS = probability of alleles identical by state, IBG = probability of SNP loci identical by genotype , NSG = probability of non-shared genotypes that have no common alleles between two genotypes.
