# Supplementary material for: Genomic Inbreeding and Relatedness in Wild Panda Populations
Source: PLoS One. 2016 Aug 5;11(8):e0160496. doi: 10.1371/journal.pone.0160496 (PMC4975500; doi:10.1371/journal.pone.0160496)
Supplement: S4 Table — (PDF) [file pone.0160496.s007.pdf]

S4 Table. Correlations between geographical distances and measures of genomic relatedness and similarity.

		IBD	d_{jk}-IV	IBS	IBG	NSG	km
f_{jk}-IV	r	0.6986	0.4572	0.9639	0.9769	0.8081	-0.6037
	p	<.0001	<.0001	<.0001	<.0001	<.0001	<.0001
IBD	r		0.7522	0.6406	0.7242	0.4130	-0.1646
	p		<.0001	<.0001	<.0001	<.0001	<.0001
d_{jk}-IV	r			0.3652	0.5206	0.1474	0.0858
	p			<.0001	<.0001	0.0005	0.0423
IBS	r				0.9765	0.6912	-0.6860
	p				<.0001	<.0001	<.0001
IBG	r					0.6741	-0.5854
	p					<.0001	<.0001
NSG	r						-0.5620
	p						<.0001

r = correlation, p = p-value, and all correlations with p<0.0001 are statistically significant with the Bonferroni multiple testing correction. f_{jk}-IV and d_{jk}-IV are genomic coancestry coefficients and dominance relationships calculated by Definitions IV of genomic relationships, respectively. IBD = probability of identity by descent, IBS = probability of identity by state, IBDG= probability of identity by genotype, NSG = probability of non-shared genotypes. km = kilometers between two locations of panda DNA sample collection.
