# Supplementary material for: Genomic Inbreeding and Relatedness in Wild Panda Populations
Source: PLoS One. 2016 Aug 5;11(8):e0160496. doi: 10.1371/journal.pone.0160496 (PMC4975500; doi:10.1371/journal.pone.0160496)
Supplement: S1 Text — (PDF) [file pone.0160496.s008.pdf]

S1 Text. Expected and observed values of probability of identify by state (IBS), probability of identity by genotype (IBG) and probability of non-shared genotypes (NSG) for identifying outliers of observed values.

As shown in Equations 5-7 of the main text, IBS, IBG and NSG are functions of allele frequencies. Let p = allele frequency of allele *A*, and q = 1−p = allele frequency of allele *a*. Then, IBS and IBG reach their lower bound whereas NSG reaches its upper bound at p = q = 0.5, as shown by the figure below, where IBS_e, IBG_e and NSG_e were the expected IBS, IBG and NSG values calculated using Equations 5-7 respectively.

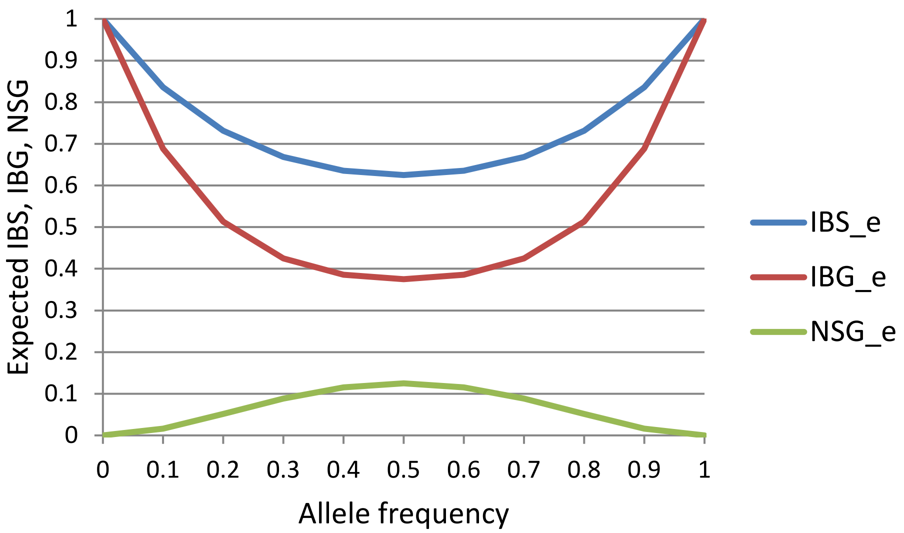

The comparison of the expected boundary values of IBS, IBG and NSG with their observed boundary values offers an assessment of the quality of the SNPs. An agreement between the expected and observed boundary values is an indication of good SNP quality. The expected boundary values of IBS, IBG and NSG were calculated using the observed boundary values of minor allele frequency (MAF) in Equations 5-7 in the main text, with MAF = 0.102 being the observed lower bound of MAF and MAF = 0.5 the upper bound of the 150K SNP set. The observed boundary values were the highest and lowest values calculated for all pairs of the forty-nine pandas using 150K SNP set. As shown by the results in the table below, all the observed boundary values of IBS, IBG and NSG were within their theoretical boundaries except one outlier of IBS and IBG involving the same pair of pandas (GP39 and GP31). We interpret this result as indication of excellent quality of the 150K SNP set in this study.

	IBS	IBG	NSG
Expected value at p = 0.102	0.834	0.375	0.017
Expected value at p = 0.500	0.625	0.684	0.125
Observed boundary values	0.645-0.824<br>Outlier: 0.925	0.441-0.679<br>Outlier: 0.861	0.047-0.108
